# Computational approaches for predicting variant impact: An overview from resources, principles to applications

**DOI:** 10.3389/fgene.2022.981005

**Published:** 2022-09-29

**Authors:** Ye Liu, William S. B. Yeung, Philip C. N. Chiu, Dandan Cao

**Affiliations:** ^1^ Shenzhen Key Laboratory of Fertility Regulation, Reproductive Medicine Center, The University of Hong Kong-Shenzhen Hospital, Shenzhen, China; ^2^ Department of Obstetrics and Gynaecology, Li Ka Shing Faculty of Medicine, The University of Hong Kong, Hong Kong SAR, China

**Keywords:** *in silico* prediction, human genetics, genotype-phenotype relationship, nonsynonymous variants, variant impact

## Abstract

One objective of human genetics is to unveil the variants that contribute to human diseases. With the rapid development and wide use of next-generation sequencing (NGS), massive genomic sequence data have been created, making personal genetic information available. Conventional experimental evidence is critical in establishing the relationship between sequence variants and phenotype but with low efficiency. Due to the lack of comprehensive databases and resources which present clinical and experimental evidence on genotype-phenotype relationship, as well as accumulating variants found from NGS, different computational tools that can predict the impact of the variants on phenotype have been greatly developed to bridge the gap. In this review, we present a brief introduction and discussion about the computational approaches for variant impact prediction. Following an innovative manner, we mainly focus on approaches for non-synonymous variants (nsSNVs) impact prediction and categorize them into six classes. Their underlying rationale and constraints, together with the concerns and remedies raised from comparative studies are discussed. We also present how the predictive approaches employed in different research. Although diverse constraints exist, the computational predictive approaches are indispensable in exploring genotype-phenotype relationship.

## 1 Introduction

One of the primary goals of human genetics is to discover the genetic variants associated with the onset and progression of human disease. The challenge is a “a needle in haystack” problem: how to pinpoint the potential causative ones from millions of individual variants (Genomes Project et al., 2015) spreading over the newly assembled, non-gap 3.055 billion–base pair human genome sequence (Nurk et al., 2022). Efforts to achieve this goal, such as linkage analysis and genome-wide association studies, were inadequately effective in identifying causative candidates and had poor clinical predictive value (Tam et al., 2019).

Over the last decade, the next generation sequencing (NGS) has been extensively utilized in biomedical research as consequences of its substantially reduced cost and generation of large volume of data. According to the fact sheets on genomic cost provided by the National Human Genome Research Institute (NHGRI) (KA., 2021), NGS technology achieved one hundred-fold cost reduction compared to Sanger sequencing, and the price is currently less than $1,000 per human genome. Nowadays, NGS platforms can finish one run within 2 days producing billions of reads for up to 48 samples (Hu et al., 2021). With the raw NGS data, standard and well-recognized variant format files can be generated using upstream analysis pipeline (Kanzi et al., 2020). Whereas the downstream disease-causing variant fishing step among ∼50,000 variants from WES, or even millions of variants from WGS is the most challenge part (Eberle et al., 2017; Koboldt, 2020).

There are plenty of data resources storing evidenced genotype-phenotype relationship information. To a certain extent, clinicians and researchers are able to utilize these records to interpret the formation, progress, diagnosis and treatment of diseases from a genetic perspective. However, even the most well-recognized databases, such as ClinVar (Landrum et al., 2020), only contain around 14,000 of highly confident variants with evidence evaluated by genetic experts, which is a small fraction compared to the huge number of variants identified from NGS. This situation dramatically reduces clinical utility from genetics. In addition, it also poses great challenges for understanding differential actions of genes between/among individuals, populations and species, as well as deciphering the genotype-phenotype relationship (Orgogozo et al., 2015). To address these issues, computational tools for predicting variant impact have emerged which can help bridge the gap between vast amount of genomic data generated and limited known genetic evidence, and finally build up the potential genotype-phenotype relationship for the newly identified variants.

Variant call format (VCF) files store identified variants providing variant genomic position, nucleotide substitution, assessed quality score, genotype and other relevant information according to alignment and variant calling information (Danecek et al., 2011). Based on the specified information, variant annotation can locate them to specific genes or transcripts, classify them into different types and conclude on their impactable consequences (Wang et al., 2010; Cingolani et al., 2012; McLaren et al., 2016). Variants causing sequence alteration are mainly categorized into four types: insertion, deletion, single nucleotide variant (SNV) and other substitution, including multiple nucleotide variant (MNV) (Eilbeck et al., 2005). Among them, SNVs are the most frequently identified (Genomes Project et al., 2015; Lek et al., 2016) and annotated (Cunningham et al., 2015). SNVs are composed of non-synonymous SNVs (nsSNVs) and synonymous SNVs (sSNVs). Comparing to sSNVs, nsSNVs, which will cause amino acid change based on the protein translation codons, are estimated at higher frequency in individuals with excess deleteriousness (Genomes Project et al., 2012). Therefore, in this review, we focus on the computational approaches which are developed to infer the impact of nsSNVs in coding regions. The database resources that are utilized by majority of the predictive methods (we name them as predictors throughout this review) are firstly introduced. Following that, we discuss the underlying motivation and constraints of those predictors with which we group them into six categories in an innovative manner. We also present their corresponding predictive performance and concerns from assessment studies. Finally, we demonstrate the application performance of the predictors in large-scale studies, as well as their ability to reveal the genotype-phenotype associations.

## 2 Database resources for variant predictors

Models are not created out of thin air; rather, they are designed to identify hidden correlations in massive volumes of real data, allowing data to be interpreted and used to generate predictions. Since the deployment of the Human Genome Project in the 1990s, various relevant databases and knowledgebases have been established and maintained by academic institutions, organizations, consortia, and communities to collect, store, and retrieve records pertaining to genetic, clinical, and phenotypic information. They provide sufficient accessible evidences and facts to reliably demonstrate the genotype-phenotype association, which explains the functional and pathogenic importance of genetic variations (Johnston and Biesecker, 2013).

Databases can be categorized according to their scope, purpose, and scale. Several reviews (Thorisson et al., 2009; Brookes and Robinson, 2015; Zhang et al., 2019; Banck et al., 2021; Katsonis et al., 2022) provided comprehensive details of the content, usage, comparisons, and limitations for those databases. In this section, we briefly review the most frequently used databases (Table 1) containing sequence information, population-scale data, phenotype ontology, clinical and experimental evidence.

**TABLE 1:** Summary of resources for human genotypes and phenotypes relationships.

Type of data	Name	Full name	Techniques	Type of variants	Targeted diseases	Website	Containing entries (until writtern in June 2022)	Composition	First publication year	Last update (until writtern in June 2022)	Accessible	Publications
Protein data	Uniprot	Universal protein resource	Curated	—	General	https://www.uniprot.org/	567,483 entries in Swiss-Prot and 231,354,261 entries in TrEMBL	UniProt Knowledgebase, UniProt Reference Clusters, and UniProt Archive	1997	2 February 2021	Free	UniProt, (2021)
Protein information	UniProtKB	Uniprot Knowledgebase	Curated	—	General	https://www.uniprot.org/uniprot/	—	Swiss-Prot and TrEMBL	—	22 November 2021	Free	UniProt, (2021)
Protein sequences	UniRef	Uniprot Reference Clusters	Curated	—	General	https://www.uniprot.org/uniref/	—	UniRef100, 90, 50	—	29 November 2021	Free	UniProt, (2021)
Protein sequences	UniParc	Uniprot Archive	Curated	—	General	https://www.uniprot.org/uniparc/	—	—	—	24 March 2022	Free	UniProt, (2021)
Protein, DNA and RNA structural data	PDB	Protein data bank	Structural data from X-ray, NMR, electron microscopy	—	General	https://www.rcsb.org/	191,565 Biological Macromolecular Structures	—	1971	14 June 2022	Free	Berman et al. (2000)
Protein data with themodynamic parameters	ProThermDB	Thermodynamic Database for Proteins and Mutants	Curated	—	General	https://web.iitm.ac.in/bioinfo2/prothermdb/index.html	∼0.12 million thermodynamic data obtained for different organisms and cell lines, >32,000 entries, ∼20,000 mutations	—	1999	22 September 2021	Free	Nikam et al. (2021)
Protein data	ONGene	—	Curated	—	Cancer	https://ongene.bioinfo-minzhao.org/index.html	803 oncogenes	—	2016	—	Free	Liu et al. (2017)
Protein data	TSGene2.0	Tumor suppressor gene database	Curated	—	Cancer	https://bioinfo.uth.edu/TSGene/	1217 human tumor suppressor genes	—	2012	4 January 2016	Free	Zhao et al. (2016)
Population data	1000 Genome Project	—	WGS	SNVs, indels	General	https://www.internationalgenome.org/	Genotypes for 2,504 healthy donor samples from 26 populations	—	2008	1 October 2015	Free	Sudmant et al. (2015)
Population data	GnomAD (previously ExAC)	Genome aggregation database	WGS, WES	SNVs, indels	General	https://gnomad.broadinstitute.org/	76,156 genomes data of diverse ancestries in v3.1 and 141,456 individuals exomes or genomes data in v2	—	2014	21 January 2022	Free	Karczewski et al. (2020)
Population data	ESP	The NHLBI exome sequencing project	WES	SNVs, indels	Disease-, phenotype-related	https://evs.gs.washington.edu/EVS/	6,503 unrelated individual exom data	—	2011	23 April 2019	Free	Fu et al. (2013)
Population data	UK Biobank	—	—	—	Disease-, phenotype-related	https://www.ukbiobank.ac.uk/	49,960 exome data	—	2006	19 March 2019	Registration fee needed	—
Population data	UK10K	—	WGS, WES	—	Healthy and disease-related cohorts	https://www.uk10k.org/	Nearly 10,000 individuals in UK population	Whole genome, Neurodevelopment, Obesity, Rare Diseases Sample Sets	2010	1 October 2015	Access control	Consortium et al. (2015)
Phenotype and genotype data	OMIM	Online Mendelian Inheritance in Man	Classification	—	Disease-, phenotype-, gene-related	https://www.omim.org/	26,446 entries, including all known mendelian disorders and over 16,000 genes	—	1960	27 May 2022	Free	Amberger et al. (2019)
Phenotype and genotype data	Orphanet	The portal for rare diseases and orphan drugs	Classification	—	Disease-, phenotype-related	https://www.orpha.net/	6,172 disease, 5835 genes	—	1997	31 May 2022	Free	—
Ontology	HPO	Human phenotype ontology	Classification	—	Disease-, phenotype-, gene-related	https://hpo.jax.org/	>13,000 terms, > 156,000 annotations	—	2008	14 April 2022	Free	Kohler et al. (2021)
Ontology	GO	Gene ontology	Classification	—	Gene-specific	http://geneontology.org/	7,510,543 annotations	Molecular Function, Cellular Component, and Biological Process	2000	16 May 2022	Free	(Ashburner et al., 2000; Gene Ontology, 2021)
Ontology	Mammalian Phenotype Ontology	—	Classification	—	Phenotype-related	https://bioportal.bioontology.org/ontologies/MP/?p=summary	14,716 classes	—	2005	14 June 2022	Free	Smith et al. (2005)
Genomic data	HGMD	Human gene mutation database	Curated	SNVs, indels	Disease-, phenotype-related	http://www.hgmd.cf.ac.uk/ac/index.php	352,731 mutation entries	352,731 mutation entries	1996	31 May 2022	Registration needed	Maffucci et al. (2019)
Genomic data	VariBench	A benchmark database for variations	Curated	SNVs, indels	—	http://structure.bmc.lu.se/VariBench/index.php	—	VariBench datasets include disease-causing missense variations, neutral high frequency SNPs, protein stability affecting missense variations, variations affecting transcription factor binding sites, variations affecting splice sites	2012	—	Free	Sasidharan Nair and Vihinen, (2013)
Genomic data	VariSNP	—	Curated	SNVs, indels	—	http://structure.bmc.lu.se/VariSNP/index.php	145,435,955 variants	Datasets selected from dbSNP which were filtered for disease-related variants found in ClinVar, Swiss-Prot and PhenCode	2014	16 February 2017	Free	Schaafsma and Vihinen, (2015)
Genomic data	dbSNP	Single nucleotide polymorphism database	Curated	SNVs, indels, retroposable element insertions and microsatellite repeat variations	General	https://www.ncbi.nlm.nih.gov/snp/	1,085,850,277 refSNP	—	1999	26 May 2020	Free	Sherry et al. (2001)
Genomic data	ClinVar	—	Curated	SNVs, indels	Disease-, phenotype-, gene-related	https://www.ncbi.nlm.nih.gov/clinvar/	1,540,318 unique variation records	—	2013	5 May 2022	Free	Landrum et al. (2020)
Genomic data	ClinGen	—	Curated	SNVs, indels	Disease-, phenotype-related	https://clinicalgenome.org/	Unique 3692 variants in unique 2278 genes	—	2013	1 April 2022	Free	Rehm et al. (2015)
Genomic data	DoCM	Database of Curated Mutations	Curated	SNVs, indels	Cancer	http://www.docm.info/	1,364 variants among 122 disease type	—	2014	—	Free	Ainscough et al. (2016)
Genomic data	VKGL	Vereniging klinisch genetische	Curated	SNVs, indels	Disease-, phenotype-related	https://vkgl.molgeniscloud.org/	188,502 variants	—	2018	December 2021	Free	Fokkema et al. (2019)
Genomic data	CIViC	Clinical interpretation of variants in cancer	Curated	SNVs, indels, SVs	Cancer	https://civicdb.org/welcome	3165 variants, 470 genes with clinical interpretation	—	2015	1 May 2022	Free	Griffith et al. (2017)
Genomic data	COSMIC	Catalogue of somatic mutations in cancer	Curated	SNVs, indels	Cancer	https://cancer.sanger.ac.uk/cosmic	29,399,170 variants, 1,207,190 CNVs, 19,422 fusions	—	2004	31 May 2022	Free	Tate et al. (2019)
Genomic data	LOVD3.0	Leiden open variation database 3.0	Curated	SNVs, indels	Disease-, phenotype-related	https://www.lovd.nl/3.0/home	800,780 variants	—	2002	17 August 2021	Free	Fokkema et al. (2021)
Genomic data	InSight	The International Society for Gastrointestinal Hereditary Tumours	Curated	SNVs, indels	Gene-specific	http://insight-database.org/	35,644 variant entries from 9 genes related to gastrointestinal tumours	Variants are automatically sourced from LOVD3	2005	—	Free	Fokkema et al. (2021)
Genomic data	HuVarBase	Human variants database	Curated	Missense, nonsense, insertion, deletion	Disease-, phenotype-related	https://www.iitm.ac.in/bioinfo/huvarbase/index.php	774,863 variants from 18,318 proteins, including 702,048 disease-causing and 72,815 neutral variants	Sources from 1000 Genomes, ClinVar, COSMIC, Humsavar, SwissVar, MutHTP, PROXiMATE	2018	15 October 2018	Free	Ganesan et al. (2019)
Genomic data	DVD	Deafness variation database	Curated	SNVs, indels	Deafness-related	https://deafnessvariationdatabase.org/	223 genes	Sources from ClinVar, dbNSFP, gnomAD, VEP, CADD, dbSNP, Population Analysis and others	2018	4 January 2021	Free	Azaiez et al. (2018)
Genomic data	METABRIC	Molecular Taxonomy of Breast Cancer International Consortium	Targeted NGS	SNVs, indels	Breast cancer	Mutation details can be retrived from https://www.cbioportal.org/study/summary?id=brca_metabric	Mutation data in 173 genes from 2433 primary breast tumor samples and 650 normal controls	Genomic mutation data, copy number aberration (CNA), gene expression and long-term clinical follow-up data	2012	—	Free	(Curtis et al., 2012; Pereira et al., 2016)
Genomic data	TCGA-BRCA	—	WES	SNVs, indels	Breast cancer	https://portal.gdc.cancer.gov/projects/TCGA-BRCA	Mutation data from WES of 817 Breast Invasive Carcinoma tumor/normal pairs	Genomic mutation data, copy number aberration (CNA), gene expression and long-term clinical follow-up data	2012	8 October 2015	Free	Ciriello et al. (2015)
Genomic data	*BRCA1* dataset	—	Saturation genome editing assays	SNVs	*BRCA1* gene	https://sge.gs.washington.edu/BRCA1/	3,893 SNVs located within or near 13 exons that encode for the RING and BRCT domains of BRCA1 (exons 2–5 and 15–23, respectively)	—	2018	—	Free	Findlay et al. (2018)
Genomic data	VarCards	—	Curated	SNVs, indels	General	http://varcards.biols.ac.cn/	110,154,363 SNVs, and 1,223,370 indels in coding regions or splicing sites	Variant-level and gene-level resources	2016	28 June 2020	Free	Li et al. (2018)

### 2.1 Sequence resources

GenBank (Sayers et al., 2022), hosted by National Institutes of Health (NIH), European Nucleotide Archive (ENA) (Baker et al., 2000), hosted by European Molecular Biology Laboratory’s European Bioinformatics Institute (EMBL-EBI), as well as the DNA Data Bank of Japan (DDBJ) (Okido et al., 2022) are the most widely used sequence databases, storing over 2.5 billion nucleotide sequences for over 504,000 formally described species. They serve as a basis for genetic analysis since aligning clean reads to the reference genome is an indispensable step in NGS analysis. As sequences of plethora species become accessible, protein sequences with 100%, 95%, and 50% identity are assembled to create clusters that are stored in informative databases such as UniProt Reference Clusters (UniRef) (Suzek et al., 2007), a branch of Universal Protein Resource (UniProt) (UniProt, 2021). These clusters are utilized to build multiple sequence alignment (MSA) sets, which form the basis of homology sequence-based approach.

### 2.2 Population resources

Several worldwide population projects exist, including the NCBI dbSNP (Smigielski et al., 2000), 1000 genome project (1KGP) (Sudmant et al., 2015), HapMap (International HapMap, 2003), UK10K (Consortium et al., 2015), Genome Aggregation Database (gnomAD) (Karczewski et al., 2020), and NHLBI GO Exome Sequencing Project (ESP) (Fu et al., 2013). With their progress and completion, their reports are now public and offer an exquisite view of the landscape of human genetic variants ranging from common to extremely rare ones. They also provide valuable information allowing the examination of variants between and within subpopulations with different ethnicities or disease status like heart, lung and blood disorders. Furthermore, minor allele frequency (MAF) from these databases is usually a useful indicator for prioritization or pertain as important feature for building prediction models.

### 2.3 Phenotype resources

Phenotype databases describe phenotypes and illnesses in conjunction with genetic information. The most widely known are OMIM (Online Mendelian Inheritance in Man) (Amberger et al., 2019) and Orphanet (Ayme et al., 1998). Their goal is to offer high-quality information on common and rare diseases or phenotypes in order to comprehensively review the genotype-phenotype association. To assist the investigation on connections between phenotypes and genes and to describe diseases in an algorithm-friendly data structure, ontology databases such as Human Phenotype Ontology (HPO) (Robinson et al., 2008), Mammalian Phenotype Ontology (Smith et al., 2005), and Gene Ontology (GO) (Ashburner et al., 2000) were developed. They are designed to annotate clinical phenotypes and genes with well-structured, computational-friendly, precise, and accurate terminology. Overall, these databases provide valuable insights for prioritization and interpretation of genetic data.

### 2.4 Clinical genetic resources

Several databases curated genetic data with clinical significance information. These databases are also known as Locus-Specific Databases (LSDB). Data and entries are often curated from literature and clinical trials. LSDBs range in scale from a single gene with roughly 4000 variants (Findlay et al., 2018) to hundreds of millions of variants (Schaafsma and Vihinen, 2015). The goal of LSDBs is to unambiguously and accurately define and categorize genotype-phenotype correlation, to understand gene functions and effects, to provide a map of genetic distribution across populations and diseases, and to assist clinicians/diagnostic laboratories in conducting further validation assays by providing detail molecule, pathogenicity, and effects of variants (Greenblatt et al., 2008). A well-curated and annotated LSDB is a valuable resource for constructing and evaluating prediction models. But note in mind that there would be overlapped variants in different LSDBs, even with contradictory classification of clinical impact due to inconsistent rules and subjective opinion of different curators. Phenotype-/disease- specific LSDBs are established, such as DVD (Deafness Variation Database) (Azaiez et al., 2018) for deafness, RAPID (Resource of Asian Primary Immunodeficiency Diseases) (Keerthikumar et al., 2009) for primary immunodeficiency disease, InSiGHT (The International Society for Gastrointestinal Hereditary Tumors) (Fokkema et al., 2021) for gastrointestinal tumors, fabry-database.org (Saito et al., 2011) for Fabry disease. Thanks to the effort of the Leiden Open source Variation Database (LOVD) (Fokkema et al., 2021) platform, a comprehensive list of public LSDBs are presented with details for researchers and clinicians to retrieve gene and mutation information from different resources.

## 3 Various variant predictors

Each predictor has a unique biochemical or biological basis. It is important to remember that the outcome of the predictor on different bases has different implications. The terms “dangerous,” “pathogenic,” “conservative,” and “damaging” do not necessarily denote causal of a specific phenotype or condition. Knowing the principles and drawbacks of each type of predictor aids in correctly interpreting the variants.

**FIGURE 1:**
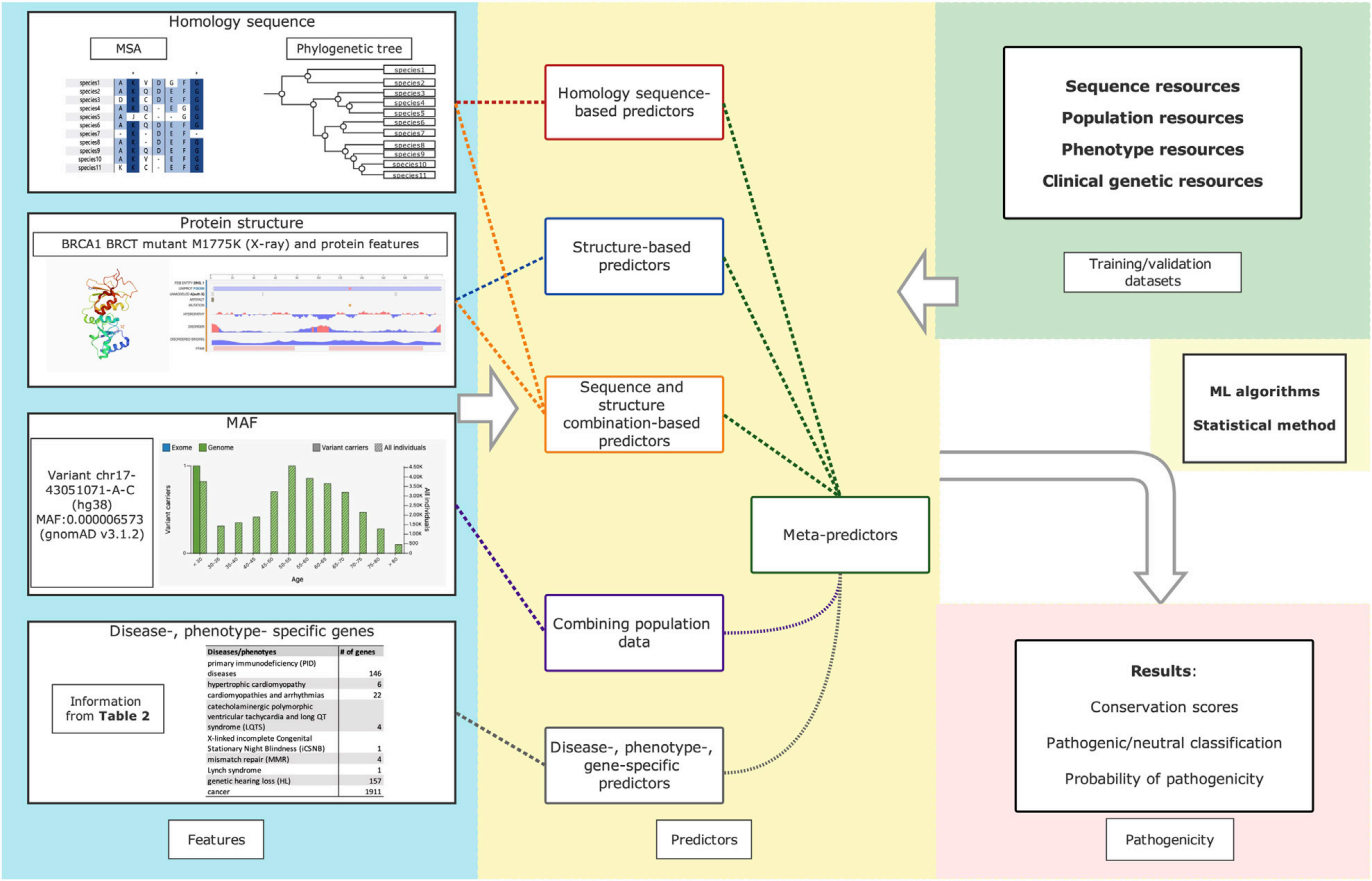
Summarized workflow of variants impact predictors. Protein structure and protein features of BRCA1 BRCT mutant M1775K are retrived from studies. (Birrane, 2006: Tischkowitz et al., 2008). The minor allele frequency (MAF) information of variant rs41293463 (chr17-43051071-A-C(GRCh38)) was retrived from gnomAD (Genome Aggregation Database).

Variant impact predictors can be categorized in different ways: machine-learning (ML) and non-ML models based on the used algorithms; homology sequence-based and structural-based models regarding the features they used in prediction; supervised and unsupervised ML-models. Unlike the category of sequence-, structure- and meta-methodologies in other reviews (Hassan et al., 2019b; Yazar and Ozbek, 2021), we introduced an innovative category here based on the characteristics and included features of each type (Figure 1). We discuss these categories by outlining the rational reasoning behind the predictors and provide an overview of the constraints. Later, we discuss predictor performance evaluation and underline current concerns and remedies. Details for each tool are present in Supplementary Table S1 and Table 2.

**TABLE 2:** Representative diseases-, phenotypes-, genes-specific variants impact predictors.

Characteristic category	Name	Type of variants	Targeted disease/phenotype/gene	# of genes	Website	Distribution (web-server/stand-alone)	First publication	Programming language	Algorithm/model	Features	Dataset for modeling	Classification index	Classification	Additional data	Publication
Meta-predictor	VIPPID (Variant Impact Predictor for PIDs)	Missense	Primary immunodeficiency (PID) diseases	146	https://mylab.shinyapps.io/VIPPID/	Web and stand-alone	April 2022	Perl, R	Conditional Inference Forest	85 features including AA, exonic, protein structural, conservation, and 20 pre-existing prediction tools	4,865 disease-associated variants from Asian Primary Immunodeficiency Diseases (RAPID) database, HGMD and ClinVar; 4,237 neutral variants from gnomAD	Classifier	Pathogenic/non-pathogenic	26 reviewed P/LP variants of known PID pathogenic genes from 1318 patients cohort and 39 validated in-house variants	Fang et al. (2022)
Meta-predictor	CanPredict	Missense	Cancer	—	http://www.canpredict.org/or http://www.cgl.ucsf.edu/Research/genentech/canpredict/, both are not accessible	—	May 2007	R	RF	SIFT, Pfam-based LogR.E-value and GO Similarity Score (GOSS) metrics	—	Classifier	Likely cancer/likely non-cancer/not determined	—	Kaminker et al. (2007)
Meta-predictor	PolyPhen-HCM	Missense	Hypertrophic cardiomyopathy	6	http://genetics.bwh.harvard.edu/hcm/	Pre-computed results	February 2011	—	Naïve bayes classifier	Prediction scores, protein structure comparison score	74 curated variants from literitures and manually classified by Laboratory for Molecular Medicine standard variant-assessment pipeline (41 pathogenic, 26 benign)	Classifier	Pathogenic/benign/no call	—	Jordan et al. (2011)
Meta-predictor	Cadioboost	Missense	Cardiomyopathies and arrhythmias	22	https://www.cardiodb.org/cardioboost/	Pre-computed results	October. 2020	R	2 Adaptive Boosting (Adaboost) classifiers	76 functional features	CM datasets: 356 rare P/LP variants from 9,007 clinical CM patients, 302 rare missense variants in CM genes from 2,090 healthy controls. Inherited arrhythmia dataset: 252 P/LP in arrhythmia-associated genes from ClinVar, 237 rare missense variants in arrhythmia genes from 2,090 healthy controls	Pathogenicity score	Disease-causing/VUS/Benign	4 datasets from ClinVar, HGMD, Oxford Medical Genetics Laboratory (OMGL), a large registry of HCM patients, SHaRe	Zhang et al. (2021)
Multiple features	GENESIS (GENe-specific EnSemble grId Search)	Variants of uncertain clinical significance	Catecholaminergic polymorphic ventricular tachycardia and long QT syndrome (LQTS)	4	https://github.com/rachellea/medgenetics	Stand-alone and pre-computed results	March 2022	Python	Logistic regression and multilayer perceptron model	8 kinds of features including AA features, domain, conservation, rate of evolution, signal-to-noise ratio, and a position-specific scoring matrix (PSSM) score	717 pathogenic variants and 3,164 benign variants curated from literiture	Probabilities of pathogenicity	Pathogenic/VUS/benign	925 VUS classified according to ACMG	Draelos et al. (2022)
Multiple features	CACNA1F-vp	Missense	X-linked incomplete Congenital Stationary Night Blindness (iCSNB)	1	https://github.com/shalawsallah/CACNA1F-variants-analysis	Stand-alone	April 2020	Python	Logistic regression model	Variant-level features and structural features	72 disease-implicated from HGMD or MGDL database, 322 benign variants from gnomAD	Probabilities of pathogenicity	Pathogenic/benign	-	Sallah et al. (2020)
Optimized PON-P2	PON-MMR2	AA substitution	Mismatch repair (MMR)	4	http://structure.bmc.lu.se/PON-MMR2/	Web and stand-alone	September 2015	R	RF	5 features: sequence conservation, physical and biochemical properties of AA	109 pathogenic, 99 neutral, 354 VUS from InSiGHT database and VariBench	Probabilities of pathogenicity	Pathogenic/VUS/benign	354 VUS dataset	Niroula and Vihinen, (2015)
Optimized MAPP	CoDP (Combination of Different Properties of MSH6 protein)	Missense	Lynch syndrome (LS)	1	http://cib.cf.ocha.ac.jp/CoDP/	Web	April 2013	—	Logistic regression model	MSA, phylogenetic tree, structral properties, MAPP, SIFT, PolyPhen2	294 missense variants from InSiGHT, MMRUV, UniProt, dbSNP, ESP, HapMap Project, 1KGP and literature	Probabilities of pathogenicity	Likely LS/Unlikely LS	260 unclassified variants dataset	Terui et al. (2013)
Meta-predictor with MAF as features	DvPred	nsSNVs	Genetic hearing loss (HL)	157	https://github.com/WCH-IRD/DVPred/tree/main/DVPred_score	Stand-alone and pre-computed results	February 2022	Python	Gradient boosting decision tree (GBDT)	65 features include conservation scores, prediction scores, MAF, gene intolerance scores and other features	1,318 P/LP and 4,628 B/LB from China Deafness Genetics Consortium (CDGC), Deafness Variation Database (DVD), ClinVar, HGMD	DvPred score	Deleterious/neutral	463 pathogenic and 454 benign variants from new version of CDGC and ClinVar	Bu et al. (2022)
Meta-predictor	NBDriver	Missense	Cancer	58	https://github.com/RamanLab/NBDriver	Stand-alone	May 2021	Python	RF, extra tress (ET) classifier, generative KDE classifier	3 types of features: one-hot encoding, overlapping k-mers, 27 genomic features	5,265 disease-associated variants from five literatures	Classifier	—	—	Banerjee et al. (2021)
Combination of rule-based and meta-predictor	CancerVar	Exon variants, CNVs, indels	Cancer	1911	https://cancervar.wglab.org/index.php	Web, stand-alone and pre-computed results	May 2022	Python	Semi-supervised generative adversarial network used in scoring method OPAI	12 clinical evidence prediction scores and 23 precomputed scores by other computational tools	13 million variants from 7 cancer knowledgebases	OPAI score	Oncogenic/benign	4 datasets from OncoKB and CIViC, IARC and literatures	Li et al. (2022)

*VUS, variant of uncertain significance.

### 3.1 Types of tools and their principles

#### 3.1.1 Homologous sequence-based predictors

This class of predictors are derived from comparative genomics. The assumption is straightforward: under natural selection, amino acid changes in conservative sequences are more “deleterious” determined by homologous sequence searching across species, than that happened in other non-homologous positions which would be deemed as “tolerant” (Cooper and Shendure, 2011). Methodologically, these predictors firstly construct the multiple sequence alignment (MSA) either by grouping multiple protein sequences with a given similarity from BLAST alignment (Altschul et al., 1990), or just retrieval customed selective sequences from afroed-mentioned genomic databases (Section 2.1) for multiple alignment using MULTIZ (Blanchette et al., 2004), or MUSCLE (Edgar, 2004). Based on MSA, a position-specific scoring matrix (PSSM) (Gribskov et al., 1987) is computed to generate the prediction outcome with probability score (Ng and Henikoff, 2001), likelihood ratio (Chun and Fay, 2009), the average distance between targeted species and others in subfamilies (Choi et al., 2012), or the entropy difference (Reva et al., 2007; Hopf et al., 2017). The predictive outcomes are normally continuous values with the designer’s recommended threshold validated in mutation datasets.

Apart from computing scores using empirically rational equations, ML algorithms were commonly utilized as classifiers. Classical models include random forest (RF) (Capriotti et al., 2006), and hidden Markov Model (HMM) (Thomas et al., 2003; Siepel et al., 2005; Garber et al., 2009; Pollard et al., 2010; Shihab et al., 2013). Although they are both ML techniques, the attributes they employ are distinct. For example, PhD-SNP (Capriotti et al., 2006) converted MSA and mutation to a 40-feature variables in support vector machine (SVM). The 40 features are composed of two parts: the first 20 vectors explicitly define the mutation residues, with -1 for the wild-type residue, 1 for the mutation, and 0 for the others. The second set of 20 vectors represents the mutation sequence environment, which is the frequency of each 20 amino acid residue in a 20 amino acid length window centered on the targeted site. Unlike unweighted and balanced MSA, HMM is a probabilistic profile of MSA that captures position-specific information (Krogh et al., 1994). Two different configurations of HMM were observed. One assumed three hidden states: “match,” “insertion,” and “deletion” to build a profile-HMM MSA (Thomas et al., 2003; Shihab et al., 2013), while the other considered a two-hidden state as “conserved” and “non-conserved” according to the phylogenetic information from tree topologies (Siepel et al., 2005; Garber et al., 2009; Pollard et al., 2010).

More recently, a novel unsupervised ML model is utilized to discover patterns and correlations between absolute locations in the MSA, allowing direct observation of both conservation and coevolution (Riesselman et al., 2018; Frazer et al., 2021). This deep generative model captured the latent structure from MSA using Variational Autoencoders (VAEs), which was proved to be an outstanding model for separation of β-lactamase protein family, at the phyla level (Detlefsen et al., 2022). By assuming the observed data *s* are generated from latent variable *z*, the decode part of VAE consists of modeling the conditional probability. Hence, the encode part is the neural network modeling of approximate posterior distribution (Riesselman et al., 2018; Frazer et al., 2021).

ML models’ predictions were normally given as log odds ratio scores between the probabilities of “substitution” and “wild-type” or “conserved” and “non-conserved”. In other words, under wild-type or neutral model, higher scores represent higher probability of unexpected substitution, thus are more evolutionary constraint.

There are two considerations regarding homologous sequence-based predictions (Eilbeck et al., 2017). Firstly, many known disease-causing alleles reside in poorly or non-conserved regions will be false-negatively classified as neutral by predictors. Secondly, the tools are inadequate for predicting stop-gain and frameshift variations since they are not included in other organisms in the MSA (Eilbeck et al., 2017). The stop-gain and frameshift variants are rated as “HIGH” impact on biological sequence in annotation tools, e.g., VEP (McLaren et al., 2010) and SnpEff (Cingolani et al., 2012). But the impact on protein is not always concordant. The amino acid changes seem to be tolerant especially the ones located near C-terminal of protein (MacArthur et al., 2012). Some frameshift variants, even in homozygous state, were frequently observed among population suggesting limited impact on human health (Eilbeck et al., 2017). Thus, additional information such as protein structure might help improve the predictive power and efficiency of the predictors, which will be discussed in the following subsections in more detail.

#### 3.1.2 Structure-based predictors

Apart from the primary structure of protein, the folding and stability are also essential for protein function normally. Early findings of variants that affect protein structure leading to aberrant phenotypes can be dated back to the 1950s, when the amino acid substitution in the half molecule of hemoglobin was discovered to cause sickle cell anemia (Ingram, 1957). Since then, thousands of mutations (Giardine et al., 2014) were described to impact on the function (increase (Jones et al., 1979) or decrease (Bonaventura and Riggs, 1968) oxygen affinity), stability (Martinez et al., 1977) and conformation (Moo-Penn et al., 1988) of hemoglobin. Indeed, missense variants also affect protein expression (Haraksingh and Snyder, 2013), post-translational modification (Kim et al., 2015) or binding affinity (Pires et al., 2015; Morningstar-Kywi et al., 2021).

An estimation of ∼75% disease-causing variants directly lead to protein destabilization, making protein stability the major contributor to disease pathology (Yue et al., 2005), whereas ∼7% variants in disease dataset also have functional role (Yue et al., 2014). The location of the mutation has a preference. In comparison to polymorphisms, disease-causing mutations predominantly impact the core of the protein, whereas ∼70% are found in structural and functionally essential regions (Sunyaev et al., 2000; de Beer et al., 2013). Protein-protein interfaces are hot spots for disease-causing nsSNVs (David et al., 2012; Petukh et al., 2015). Again, disease-causing variations were 49% more likely (interface core vs interface rim odds ratio (OR) 1.49, 95% CI 1.24–1.80, *p* < 0.00001) to be found in the interface core than in the rim, possibly due to their differences in energy contribution to protein stability, physicochemical and evolutionary properties (David and Sternberg, 2015).

Typically, nsSNVs impact on protein stability is estimated by computing the variation of Gibbs free energy change (∆∆G_f_) resulting from an amino acid substitution. Physical effective energy function, statistical potential function, and empirical defined potential function are the three types of energy computing methodologies (Guerois et al., 2002). Because the first function is computationally intensive, the latter two are more frequently utilized. Structure-based predictors of protein stability mainly attribute to empirical potentials that integrate physical and statistical structure-related energy components (Guerois et al., 2002), and ML techniques (Dehouck et al., 2009; Laimer et al., 2015).

In theory, these approaches should potentially give greater insights into the mutation effect than the homologous sequence-based predictors since they are built on the direct impact of mutation on protein structure and function. However, the truth is that protein-based predictors are still limited because of the unbalance and intrinsic variability of the thermodynamic data and their prediction performance (Sanavia et al., 2020). On one hand, despite that the Protein Data Bank (PDB) (Berman et al., 2000) contains over 50,000 human protein records, many of them are redundant, covering only 70% of reference human proteome at a sequence identity level higher than 30% (Somody et al., 2017). The development of AlphaFold2 (Tunyasuvunakool et al., 2021), to an extent increases the protein structure coverage; but its capability to predict the impact of single mutation is questionable (Pak et al., 2021; Buel and Walters, 2022). On the other hand, sequence-based techniques, under certain circumstances, outperform structure-based stability prediction tools (Hoie et al., 2022). Thus, combining sequence with structural information may aid in improving prediction capacity of variant impact.

#### 3.1.3 Sequence and structure combination-based predictors

The approaches of this category consider both the previously described homologous sequence and protein structure information. Predictions take benefit from the combination of homology sequence information (e.g., conservative scores), and the structure features, such as hydropathy, polarity, backbone angles and electrostatic interactions, supplemented with energy features and biochemical features such as solvent accessible surface area of the interface (Kulshreshtha et al., 2016). Those features are sometimes transformed or selected for model training to achieve high prediction efficiency. Sometimes hundreds of features might be incorporated into the final model (Niroula et al., 2015). Algorithmically, supervised ML approaches including SVM (Calabrese et al., 2009; Li et al., 2009), naïve bayes classifier (Adzhubei et al., 2010), neural network (NN) (Hecht et al., 2015), RF (Carter et al., 2013; Niroula et al., 2015) and boosted tree regression (Zhou et al., 2016) are commonly applied in the multiple features predictors.

#### 3.1.4 Meta-predictors

Meta-predictors are tools that make predictions by integrating results of pre-existing predictors. The term “meta-” sometimes corresponds to the term “consensus” in other studies (Bendl et al., 2014). The basic idea behind meta-predictors is to leverage on potential complimentary performance of selected predictors in classifying variants.

There are mainly two improvements regarding meta-predictors comparing to aforementioned counterparts. First of all, meta-predictors give a comprehensive evaluation on the selected pre-existing tools. Each predictor has its own metric and scale making it difficult to compare across multiple predictors hindering the simultaneous usage. Meta-predictors have their own way to interpret scores from selected tools, by transforming to a comparable range as normalized scores (Bendl et al., 2014) or binary values (Gonzalez-Perez and Lopez-Bigas, 2011). In addition, meta-predictors are able to improve prediction performance by integrating prediction scores from different predictors, which allows the avoidance of bias and anti-generalization by single predictors (Kircher et al., 2014).

In terms of missing value, where partly pre-existing tools fail to predict, some meta-predictors impute them using deleterious/neutral threshold (Capriotti et al., 2013), average score (Kircher et al., 2014; Quang et al., 2015), fixed score (Quinodoz et al., 2022), the maximal pathogenic score (Jagadeesh et al., 2016), or a flexible imputation using average value of k-nearest neighbors (Ioannidis et al., 2016) and Bayesian principle component analysis (BPCA) (Dong et al., 2015). There is currently no gold standard for imputation. Although machine-learning imputation appears to be more accurate (Brock et al., 2008; Wei et al., 2018), meta-predictor builders revealed that missing values account for less than 10% of their training and testing datasets (Dong et al., 2015), making the imputation methods less significant difference.

While prediction performance studies suggested that meta-predictors surpassed other counterparts (Tian et al., 2019), concerns regarding circularity occurred, which will be discussed in Section 3.3.

#### 3.1.5 Combining population data

A polymorphism is defined as an alteration in DNA sequence found in the general population at a MAF greater than 1%. According to The American College of Medical Genetics (ACMG) and the Association for Molecular Pathology (AMP) guidelines for clinical variant interpretation, a variant with >5% MAF is considered as a stand-alone support for benign interpretation for a rare Mendelian disorder (Richards et al., 2015). This is supported by the “neutral theory”, which defines neutral variants as the ones settled in the population through random genetic drift causing neither harmful nor beneficial effect to the survival of individual organisms (Kimura, 1979). When training and validating predictors, variants with higher than specified allele frequency (e.g., 5% or 1%) from population-scale databases were usually denoted as benign or neutral. However, predictors in predicting neutral variants differ greatly in capacity and specificity. For example, PON-P2 (Niroula et al., 2015) had a 95% specificity, while the poorest predictor incorrectly categorized more than one-third of polymorphisms as disease-causing (Niroula and Vihinen, 2019). Classifying the impact of variants according to their MAF were further argued by different hypothesis including “rare variant for Mendelian disease” (Pritchard and Cox, 2002), “Common disease, common variant” (CDCV) and “Common disease/rare variant” (CDRV).

Researchers now have access to an exquisitely detailed view of the landscape of common and rare human genetic variants. Another issue that predictors should be careful with when utilizing MAF is that MAF is largely dependent on the population size and varies among subpopulations leading to population stratification (Eilbeck et al., 2017). For example, rs79444516, which is common in African population (13%), exhibited its extreme rareness in European and Asian population, with MAF <0.05%. When estimated in the mixture population, the MAF is 1.2% which will cause confounding classification. Varied MAF for the same variant because of different scales of sample size could be largely mitigated with the completion of the huge population-scale projects.

To better utilize MAF in prediction, ClinPred (Alirezaie et al., 2018), a meta-predictor using ML approach, employs MAFs from diverse populations as part of their features, instead of classifying variants based on single arbitrary MAF cutoffs. Together with feature scores from 16 pre-existing tools, ClinPred trains on clinically curated pathogenic and benign datasets and outperforms other meta-predictors when applied to datasets of rare diseases and cancer (Alirezaie et al., 2018). Therefore, MAFs from population data is capable to enhance the prediction. Similarly, more and more tools (Chennen et al., 2020; do Nascimento et al., 2020; Lai et al., 2020; Li et al., 2020) integrated MAFs as predicting features and achieved competitive performance on pathogenicity prediction.

#### 3.1.6 Disease-, phenotype-, gene-specific predictors

The ultimate goal of the variant prediction tools is to accelerate the development of precision medicine. Majority of the strategies discussed above aim to estimate disease occurrence based on the assumption that changes in protein function leads to a decrease in organismal fitness (Boucher et al., 2016). They are trained in a large-scale datasets in a genome-wide and pan-disease manner neglecting the complexity among different diseases and making the prediction results suboptimal (Dorfman et al., 2010). Therefore, with the necessity to precisely estimate the impact of variants on specific disease/phenotype, a class of disease-, gene-, phenotype-specific prediction has emerged.

The phenotype-targeting predictors range widely from common cardiac (Zhang et al., 2021), cancer (Kaminker et al., 2007), and neurodegenerative disease (Ahmed et al., 2015), to rare diseases, such as methylmalonic acidemia (Peng et al., 2019), X-linked incomplete Congenital Stationary Night Blindness (Sallah et al., 2020) and Pompe disease (Adhikari, 2019). More details are presented in Table 2. There are over 13,000 terms defined in HPO. While LSDBs provide benchmarked variant datasets, such as COSMIC, CIViC and OncoKB for cancer, which can be utilized for disease-specific predictors construction, limited datasets are available for majority of phenotypes. For a particular disease/phenotype, the training and validation datasets can be prepared by curation of variants and genes from literatures, or re-analysis of unpublished sequencing data of case-control cohorts, followed by manually classification using recognized guidelines such as ACMG/AMP. The scale of the curated databases for different diseases/phenotypes varies in gene (from one to hundreds) and variant number (from thousands to millions) which is largely dependent on the number of relevant publications.

With the curated databases, most of this category of predictors utilize ML methodology. They can be grouped into three classes. The first class overlaps with previous mentioned categories but has distinct characteristic. It includes sequence and structure combination-based (Sallah et al., 2020; Draelos et al., 2022), sharing the same strategy with previously mention predictors in Section 3.1.3, and meta-predictors (Jordan et al., 2011; Bu et al., 2022) similar to the ones discussed in Section 3.1.4. The distinct characteristic is the difference in training and validation datasets selection. Also, disease-related genes are known, making predictors capable of constructing sub-model for each gene, resulting in better prediction performance (Fang et al., 2022). The second class aims to optimize pre-existing predictor, usually sequence-based model, by re-constructing MSAs and phylogenetic tree of targeted gene(s) (Niroula and Vihinen, 2015; Fortuno et al., 2018). These predictors share the same strategy as their precursors, with distinct features selection. The third class predicts variants in a comprehensive and robust way, utilizing additional rule-based classification system. For example, CancerVar (Li et al., 2022), integrates rule-based categorization with ML-based meta-predictor scores to interpret the predicting clinical significance.

These well-calibrated and sculpted predictors demonstrate their capability in targeted sequencing disease-specific panels to the utmost (Peng et al., 2019). In contrast, their ability to generalize is then questioned. When utilizing these techniques, note in mind the key target phenotypes and genes.

### 3.2 Performance assessment of predictors

As dozens of predictors exist, choosing the appropriate one(s) becomes challenging for end users. Several assessment criteria, such as sensitivity, specificity, positive predictive value (PPV), negative predictive value (NPV), accuracy, and Matthews correlation coefficient (MCC), are commonly used to demonstrate model performance (Vihinen, 2012). The values for sensitivity, specificity, PPV, NPV, and accuracy range from 0 to 1, with higher values indicating better performance. MCC benefits from true and false positives and negatives with values on a scale of -1 to 1, with values closer to 1 indicating perfect prediction. Furthermore, a visualization measurement, receiver operating characteristics (ROC) analysis is frequently used to intuitively compare the area under the ROC curve (AUC) of multiple predictors (Vihinen, 2012). For non-intersecting curves, the AUC value closer to 1 suggests better overall performance, while a value of 0.5 indicates random and useless classification.

Most predictors, when developed, would be assessed using respective training and validation datasets presenting supreme or acceptable performance. However, evaluation using consensus datasets would be more informative for tool selection.

There are dozens of comparison studies on the performance assessment of different selected tools using different benchmark datasets. When Performance evaluation of pathogenicity-computation methods for missense variants, meta-predictors such as REVEL, Meta-SNP, generally have better performance and stronger evidence in clinical interpretation (Accetturo et al., 2020; Cubuk et al., 2021; Anderson and Lassmann, 2022). In the assessment of 23 predictors, Li et al. (Li et al., 2018) revealed that meta-predictors achieved higher AUC than others of sequence-based and structure-based predictors using the ClinVar benchmark dataset, indicating better performance of meta-predictors. However, when regarding somatic variants and *PPARG* gene benchmark datasets, meta-predictors and structure-based predictors exhibited comparable performance (AUC>0.8) (Li et al., 2018), and were superior to homology sequence-based predictors (AUC>0.7). Hassan et al. (Hassan et al., 2019a) revealed that meta-predictor which integrated 4 pre-existing prediction scores, outperformed other 8 predictors achieving ∼10%, 20%, 15% improvement in specificity, sensitivity and AUC, respectively.

The performance of different categories are not always consistent, and sometimes are contradictory. Poon’s study (Poon, 2021) on *BRCA1/2* datasets revealed that SIFT and PolyPhen2’s performance differed among genes. Meléndez-Aranda et al. (Melendez-Aranda et al., 2019) compared the performance of 6 *in silico* tools on 215 missense mutations in hemophilia B causative gene *F9*, and the results showed that the most popular tool, SIFT, was the most accurate. When applying to a somatic dataset containing 4319 somatic missense variants, the performance of SIFT was sub-optimal (Suybeng et al., 2020). As a result, it is critical to have pre-knowledge of your testing data and predictive goal when selecting appropriate tools.

In order to address the confounding situation and objectively determine the appropriate usage and accuracy of predictors, the Critical Assessment of Genome Interpretation (CAGI) (Andreoletti et al., 2019) community started their experiments in 2010. Until now, there are six editions with 63 challenges and over 50 articles released. Participants predict the phenotypic impact of unpublished genetic variants collected from experimental and clinical labs provided by CAGI. Later, independent assessors test the predictions against experimental characterized phenotypes, and the results will be presented at the CAGI conference and published in special journal issues. The challenges released include a wide range of topics, from nsSNVs to splicing variants, and from disease panels to databases including curated variants. However, the reality of the outcome is frequently far more complex than the challenges’ initial objective. Predictors with superior performance in one challenge, would fail to call the pathogenicity of variants in other datasets (Katsonis and Lichtarge, 2019; Savojardo et al., 2019). Complex gene datasets caused divergence predictions and confounding outcomes, raising concerns about the possibility of experimental mistakes as the basis of disagreement (Miller et al., 2019). All the above suggested the caution when interpreting the evaluation results.

### 3.3 Concerns of current predictors and remedies

Majority of predictors are trained, validated and tested using benchmarked sets of variants with explicit classification labels. When evaluated 10 predictors across major public databases, Grimm et al. (2015) raised concerns about “circularity” involving in the usage of predictors and conduction of comparative studies. The term “circularity” refers to the situation that same variants are recursively used in both training and evaluating models.

“Type 1 circularity” refers to the overlap between training and evaluation particularly for supervised ML-based predictors, resulting in poor generalization on new data (Grimm et al., 2015). Selecting predictions from unsupervised tools as features or filtering overlapping sets during training might assist to minimize the “type 1 circularity” during model construction (Alirezaie et al., 2018; Won et al., 2021). Furthermore, avoiding overuse of individual dataset (Vihinen, 2013; Weber et al., 2019) and choosing benchmark database which addressed overlapped issue (Sasidharan Nair and Vihinen, 2013; Sarkar et al., 2020) also helps when conducting comparative studies.

Grimm et al. (2015) observed that weighted FatHMM (Shihab et al., 2013) achieved outstanding performance in 2 datasets but severe drop in performance in subset from SwissVar. They found that the ratio of pathogenic and neutral variants in the same protein family was the key element for weighting scheme, leading to higher pathogenic score assigned to both neutral and pathogenic variants in the same gene with higher ratio (Grimm et al., 2015). This strategy made weighted FatHMM statistically successful in some datasets, but ultimately inappropriate. Therefore, they defined the “Type 2 circularity” as the circumstance in which all variants from the same gene are jointly labeled as pathogenic or neutral. To address this problem, it was suggested to use datasets with an appropriate pathogenic-to-neutral ratio and avoid genes with exclusive pathogenic or neutral variations when reporting performance (Bu et al., 2022; Quinodoz et al., 2022).

Another concern is about “collinearity,” which generally occurs with the regression models. ‘Collinearity’ refers to the circumstance in which significant correlation between two or more feature variables resulting in independent regression coefficients estimation problems and leading to redundancy in the set of variables (Bayman and Dexter, 2021). This situation might be mitigated via feature selection and estimator modification (Zheng et al., 2020; Chan et al., 2022). From another perspective, “collinearity” should not be a problem because more complicated machine learning algorithms including SVM, Random Forest, and Neural Network, can handle large-scale and multi-collinear datasets in a better way (Dong et al., 2015; Perez-Enciso and Zingaretti, 2019).

## 4 Application

In-silico approaches combined mathematical strategies with expert opinion allows researchers to analyze the biological meaning of genetic data efficiently and economically (Trisilowati and Mallet, 2012). In-silico predictors on variant effect aids in genome interpretation. The prediction-based categorization provides insight into variant characterization and prioritization.

Regards to large-scale population study, *in silico* predictors aid in variant classification for pattern overview and comparison at subpopulation level. For example, Palmer et al. (2022) subdivided missense variants by SIFT and PolyPhen2 prediction in research on bipolar disorder (BD) and revealed an obvious enrichment in ultra-rare harmful missense variation outside of confined missense areas, particularly in bipolar II disorder (BD2). This observation contrasted with the findings in schizophrenia cases (Singh et al., 2022) of enrichment within constrained missense regions. The authors speculated this signal may capture something distinct to mood disorders relative to psychotic disorders (Palmer et al., 2022).

For large-scale population*, in silico* predictors also facilitate the detection of variant-level signals under natural-selection for those living in extreme environments or with a diverse geographic distribution. Deng et al. (2019) ranked variants by calculating the functional importance score (FIS) from four *in silico* predictors. Based on the ranking of adaptive genetic variants, they revealed a seldom studied gene, *TMEM247* with a missense variant rs116983452, to be the most-differentiated functional variant identified between Tibetan and non-Tibetan populations (Deng et al., 2019). When studying non-homogeneous Taiwanese Han population, integrated selection of allele favored by evolution (iSAFE) was incorporated with the CADD functional impact score to identify 16 natural-selection signals by geographic distribution that were unambiguously localized to 5 single genes (Lo et al., 2021). Meanwhile, in the western Roma population, Font-Porterias et al. (2021) categorized missense variants based on GERP, PolyPhen2 and CADD, revealing significant difference in common deleterious variant portion between Roma and non-Roma population. Furthermore, runs of homozygozity (ROH), which are continuous homozygous regions of the DNA sequence, exhibit ancestry-specific patterns of accumulation of deleterious homozygotes.

In addition to characterization for population-level study, predictors have also been widely used for prioritization of disease-causing candidates in case-control or pedigree studies, finally leading to the identification of genotype-phenotype association. There are commonly two strategies for variant prioritization in which predictors help. Several frameworks and platforms are listed in Table 3.

**TABLE 3:** Representative prioritization frameworks and tools.

Characteristic category	Name	Type of Targeted variants*	Website	Distribution (web-server/stand-alone)	First publication	Last update	Programming language	Algorithm/modules	Input type	Dataset for modeling	Publications
User-defined rule-based	VCF.Filter	SNVs, indels	https://biomedical-sequencing.at/VCFFilter/	Web and stand-alone	July 2017	—	Java	Filter cohort, prioritize on pedigree and search variant in cohort modules	VCF files, targeted regions, cohort allele frequencies, pedigree information	—	Muller et al. (2017)
User-defined rule-based	BiERapp	SNVs, indels, CNVs, MNVs, SVs	http://bioinfo.cipf.es/apps-beta/bierapp/2.0.0/#home	Web and stand-alone	April 2014	—	HTML5 and JS	CellBase annotation, consecutive filtering strategy	Multi-sample VCF files	—	Aleman et al. (2014)
User-defined rule-based	KGGSeq	SNVs, indel, CNVs	http://pmglab.top/kggseq/	Stand-alone	January. 2012	1 January 2022	Java	5 major modules: quality control, filtration, annotation, pathogenic prediction and statistic tests	VCF files, pedigree information	7,296 disease-causing variants from OMIM and 48,089 neutral variants	Li et al. (2012); Li et al. (2017)
User-defined rule-based	VPOT (variant prioritization ordering tool)	SNVs, indel	https://github.com/VCCRI/VPOT/	Stand-alone	November. 2019	27 October 2021	Python	2 steps: prioritization of variants based on user-defined parameters, post-processing of variant priority ordered list	ANNOVAR annotated VCF or TXT files, inheritance model	—	Ip et al. (2019)
ACMG guideline based	TAPES	SNVs, indel	https://github.com/a-xavier/tapes	Stand-alone	October. 2019	—	Python	Bayesian classification framework	VCF files	—	Xavier et al. (2019)
ACMG guideline based	InterVar	SNVs, indel	https://github.com/WGLab/InterVar, http://wintervar.wglab.org/	Web, stand-alone and pre-computed results	February 2017	13 June 2022	Python	Automated or manually scoring system. Manual review and adjustment on specific criteria	Annotated or unannotated VCF files	—	Li and Wang, (2017)
ACMG guideline realted	VarFish	SNVs, indels	https://varfish-kiosk.bihealth.org/, https://github.com/bihealth/varfish-server	Web and stand-alone	July 2020	June 2022	Python	Quality control, database- and user-based annotation, filtering interface, joint filtering of multiple cases	VCF files, optional pedigree information	-	Holtgrewe et al. (2020)
Phenotype-driven	Exomiser	SNVs, indels	https://www.sanger.ac.uk/tool/exomiser/	Stand-alone	November 2015	November 2021	Java	Filtering and Prioritization based on logistical regression model. Four prioritization method include PHIVE, PhenIX, ExomeWalker, hiPHIVE.	VCF files, HPO terms, optional pedigree information	—	Smedley et al. (2015)
Phenotype-driven	eXtasy	nsSNVs	https://extasy.esat.kuleuven.be/	Web and stand-alone	September 2013	—	Ruby	RF	VCF files, HPO terms	24,454 disease-causing nsSNV from HGMD associated with 1,142 HPO terms. Control datasets: common polymophisms and rare variants from 1KGP, rare variants in in-house control samples	Sifrim et al. (2013)
Phenotype-driven	AMELIE (Automatic Mendelian Literature Evaluation)	Missense, stopgain, splicing, indels, duplication	https://amelie.stanford.edu/	Web and stand-alone	May 2020	May 2021	—	Natural language processing (NLP) and logistic regression classifier	VCF files, HPO terms	A set of 681 simulated patients using data from OMIM, ClinVar and 1KGP	Birgmeier et al. (2020)
Phenotype-driven	Phen-Gen	Missense, nonsense, splice site and indels	https://github.com/pkuerten/phen-gen	Stand-alone	September 2014	—	Perl	Random walk–with–restart algorithm, Bayesian framework based on genotype and phenotype data	VCF files, HPO terms	HGMD 2011.4 datasets	Javed et al. (2014)
Phenotype-driven	LIRICAL (LIkelihood Ratio Interpretation of Clinical AbnormaLities)	SNVs, indels	https://github.com/TheJacksonLaboratory/LIRICAL	Stand-alone	September 2020	September 2021	Java	Likelihood-ratio	VCF files, HPO terms	—	Robinson et al. (2020)
Phenotype only	Phrank (phenotype ranking)	—	https://bitbucket.org/bejerano/phrank/src/master/	Stand-alone	February 2019	—	Python	Boolean Bayesian network	HPO terms	Knowledgebase of gene-disease-phenotype relationships, HPO-A	Jagadeesh et al. (2019)
Phenotype only	PhenoRank	—	https://github.com/alexjcornish/PhenoRank	Stand-alone	June 2018	—	Python	Phenotypic similarity measured by simGIC, gene scores calculation by random walk with restart (RWR) method	HPO terms	5,685 unique associations between 4,729 diseases and 3,713 genes from ClinVar, OMIM and UniProtKB	Cornish et al. (2018)
Phenotype only	Phen2Gene	—	https://phen2gene.wglab.org/, https://github.com/WGLab/Phen2Gene	Web and stand-alone	June 2020	March 2021	Python	Weighting by skewness	HPO terms	HPO–gene annotation files downloaded from the Jackson Laboratory for Genomic Medicine; gene-disease databases OMIM, ClinVar, Orphanet, GeneReviews; gene-gene relationship databases HPRD, HGNC, Biosystem, HTRI	Zhao et al. (2020)

First, empirical criteria are used to filter variations. With high quality variants, many studies (Ma et al., 2013; Blue et al., 2014) performed prioritization based on *in silico* predictions, MAF in population database and control groups, inheritance pattern, and functional effect. By this method, less than 10 variants are distilled out of hundreds of thousands obtained from WES analysis. Following the validation of orthogonal assays (e.g., Sanger sequencing), true positive causal candidates will be examined for the functional effect on protein *in vitro* and/or *in vivo*. The relationship between variants-phenotype is therefore thoroughly investigated. Several user-friendly rule-based frameworks (Coutant et al., 2012; Li et al., 2012; Aleman et al., 2014; Muller et al., 2017) have been built to make the filtering procedure easier to implement. Researchers can set their own criteria and get the findings in readable files with detailed annotation information. The prioritization can also be supplemented with adoption of consensus recommendations, such as ACMG/AMP standards and guidelines (Richards et al., 2015). The guideline includes a comprehensive set of definitions and criteria for variation interpretation, ranging from standardized nomenclature to evidence-based rating yielding a five-tier terminology system outcome. Results from *in silico* predictors are accounted as “supporting” evidence for benign or pathogenic classification. Some automatic tools (Li and Wang, 2017; Xavier et al., 2019) have also been developed for variant classification based on the guidelines, although the manual classification by professional geneticists would be deemed as the golden standard.

The second strategy refers to phenotype-driven frameworks, which combine phenotype and variants data for prioritization and interpretation. Clinical diagnosis would be straightforward when the disease is known. However, before the identification of candidate disease, the procedure to explain a set of clinical features is challenging due to the absence or presence of unrelated features and various degrees of specificity (Kohler et al., 2009). To extract standardized and normalized phenotypic terminologies from sparse clinical abnormalities in case studies, some tools like Phenomizer (Kohler et al., 2009) and Doc2HPO (Liu et al., 2019) are recommended to map the clinical symptoms to the list of known disorders and estimate the significance of each disease match. Prediction scores from *in silico* predictors are integrated in this kind of framework as “pathogenicity” or “deleteriousness” features. Most of phenotype-driven tools (Sifrim et al., 2013; Javed et al., 2014; Smedley et al., 2015; Birgmeier et al., 2020; Robinson et al., 2020) require variant files and HPO terms as input, while some tools (Cornish et al., 2018; Jagadeesh et al., 2019; Zhao et al., 2020) require only HPO terms. Yuan et al. (2022) investigated causal-gene prioritizing performance of both types on two benchmark datasets in a recent comparative study and revealed that former ones performed better overall than latter ones. Their results also indicated the complementarity of multiple phenotype-driven tools towards a viable integrated strategy may improve diagnostic efficiency (Yuan et al., 2022).

## 5 Discussion

In this review, we firstly summarized the database resources frequently used during predictor development. We then discussed the rational, necessity and limitations for the newly categorized predictors: homologous sequence-based, structural-based, combination of sequence and structural, meta-predictors, population-based, and gene-, phenotype-, disease-specific predictors. Predictor performance as well as their limitations and possible remedies were then outlined. The application of the predictors in real studies was finally presented demonstrating their efficient assistance in variant characterization and prioritization, as well as the discovery of genotype-phenotype association.

When building predictors, unambiguous labeled datasets are critical. Avoiding overlapping and contradicting data, as well as balancing the positive-negative ratio in training and validation datasets, will definitely minimize the negative influence of circularity. Further examination on the collinearity between/among feature variables will facilitate the optimization of prediction models, even though some algorithms are literally not affected.

Among the predictors, meta-predictors outperform others in general; however, their prediction performance is considerably discounted in some disease-specific datasets, raising concern about their applications especially in clinical settings (Schiemann and Stowell, 2016; Mahmood et al., 2017). Employment of disease-, gene-, phenotype-specific predictors can to an extent solve the above issue. When selecting predictors for a particular study, efforts should be given on screening whether the genes and phenotype predictor calibrated perfectly matching your research, and understanding the scope and predictive performance of each predictor. On the other hand, we look forward to more specialized predictors sculpted for a variety of phenotypes covering both common and rare diseases.

According to Variation Ontology (VariO) (Vihinen, 2014), variant impacts on protein level can be annotated with effects on function, structure and property. Variants impact on protein functional or property effects can be classified as follows: abundance, which includes gene dosage, expression, degradation and mis-localization; activity, which includes enzymatic, kinetic and regulation; enzymatic specificity, and molecular affinity (Vihinen, 2021). Most of above-mentioned predictors computed the possibility of pathogenic effect on protein function and structure in a broad range, rather the effects on protein abundance, activity or affinity properties separately. This may indicate a challenging future orientation of variant predictors development.

The correlation between variants pathogenic prediction on protein function or structure and abnormal clinical outcomes are validated by experimental facts at the current stage. For certain phenotypes, an evident enrichment of deleterious variants in a set of disease-related genes, such as the increased mutational burden in essential genes in autism spectrum disorder (Ji et al., 2016), WNT signaling genes in myelomeningocele (Hebert et al., 2020), a set of 5 genes in epilepsy (Leu et al., 2015). The gap between observed higher burden genes and clinical phenotype is then bridged by functional or mechanical experimental studies. For example, meiocytes with pathogenic mutation p.S167L in *HSF2BP* found in premature ovarian insufficiency (POI) patients from a family, showed a reduced number of foci formed by the recombinases RAD51/DMC1, leading to crossover defect, which provided an insight into the molecular mechanism of mutation in POI and subfertility (Felipe-Medina et al., 2020). Currently, variant impact predictors are insufficient for indicating molecular mechanism of pathogenicity. However, the advancement of protein structure prediction may assist the interpretation of pathogenic variants since structural information gives useful insights in evaluating variant impact on protein or biological systems (Diwan et al., 2021).

Impacts of mutations on protein synthesis includes transcriptional and translational influences. For SNVs, the impact on transcription involves in changes in transcript sequence and influence in gene regulation (Haraksingh and Snyder, 2013). Tools for predicting impact on gene regulation have been timely and systematically reviewed by other studies (Li et al., 2015; Ohno et al., 2018; Rojano et al., 2019; Canson et al., 2020). In terms of translation, SNVs-induced amino acid substitution causes protein structure and function abnormalities, and the prediction methods have been explored in this study. The deeper association between SNVs for protein folding and post-translation modification is still being investigated.

With the development of a cutting-edge structure prediction tool, AlphaFold2, the unstructured human protein narrowed down to less than 30% (Porta-Pardo et al., 2022). However, examples showed that AlphaFold2 was not capable for predicting protein structure modification caused by pathogenic mutations, particularly those having experimentally proven destabilizing effect (Buel and Walters, 2022). The reasons for this limitation may relate to the bioinformatics and physical methodologies utilized in modeling, as well as the resources from protein sequence and PDB structure data employed, instead of the fundamental driving forces of protein folding (Jumper et al., 2021; Buel and Walters, 2022). The AlphaFold team is presently considering solutions for new mutations, which may give better prediction on unfolding to folding state, based on protein physics instead of sequence evolutionary (Callaway, 2022). We anticipate that its success will usher in a new age of human genetic research, including the acceleration of *in silico* functional and mechanical genotype-phenotype association investigations.

Finally, although the variant effect predictors greatly help the genomic interpretation, end-users should keep in mind that the predictor’s role is only an assistance to clinical diagnosis, and merely a starting point (Eilbeck et al., 2017). The unequal relationship between predicted damaging effect and pathogenicity warns their usage. In addition, under some circumstances, the predicted scores overstating the effect of uncommon mutations, will cause inflated estimation affecting the specificity and sensitivity (Lanktree et al., 2018). Therefore, experimental validations, the golden standard in variant impact evaluation, are still indispensable.
