# Supplementary material for: Computational approaches for predicting variant impact: An overview from resources, principles to applications
Source: Front Genet. 2022 Sep 29;13:981005. doi: 10.3389/fgene.2022.981005 (PMC9559863; doi:10.3389/fgene.2022.981005)
Supplement: Supplementary file 1 [file Table1.docx]

Supplementary Table 1. Representative predictors for general genomic variants impact.

| Characteristic category | Name | Full name | Type of Targeted variants | Website | Distribution (web-server/stand-alone) | First publication | Last update | Programming language | Algorithms/models | Features | Dataset for modeling * | Classification index | Classification | Cut-off | Additional benchmark data * | Publications | Times cited in total (Web of Science until writtern in June 2022) |
| --- | --- | --- | --- | --- | --- | --- | --- | --- | --- | --- | --- | --- | --- | --- | --- | --- | --- |
| Homology sequence-based | SIFT;  SIFT 4G | Sorting Intolerant from Tolerant | Missense mutations | <https://sift.bii.a-star.edu.sg/> | Web | May, 2001 | March 31, 2022 | - | Position-Specific Probability Estimation | Sequence homology | - | p_ca_ | Deleterious/ tolerated | (0,0.05);  [0.05,1] | Swiss-Prot, Swiss-Prot with Trembl, UniRef-50, UniRef-90 and UniRef-100 | (Ng and Henikoff, 2001; 2003; Sim et al., 2012; Vaser et al., 2016) | 6,966 |
| Homology sequence-based | FATHMM | Functional Analysis Through Hidden Markov Models | nsSNPs | <http://fathmm.biocompute.org.uk/index.html> | Web and stand-alone | Jan., 2013 | Jan. 30, 2014 | JS and python | HMM | MSA, phylogenetic tree | Inherited disease-causing nsSNPs from HGMD and inherited putative functionally neutral nsSNPs from UniProt | Weighted and unweighted score | Disease-associated/ neutral | Unweighted: -3.0; weighted: -1.5 | VariBench, SwissVar,  1 other sets from literature | (Shihab et al., 2013a; Shihab et al., 2013b; Shihab et al., 2014) | 907 |
| Homology sequence-based | MutationAssessor | - | Protein mutations | <http://mutationassessor.org/r3/> | Web | Aug., 2007 | Dec. 31, 2015 | - | Combinatorial entropy approach | Sequence homology | Multiple sequence alignments (MSA) of homologous seqeunces from UniProt sequence database | Functional impact score | Functional/ non-functional | (3.5,5];  (1.9,3.5];  (0.8,1.9];  [-4,0.8] | 36,000 neutral and 19,000 functional variants from Uniprot; 10,000 nsSNPs from COSMIC | (Reva et al., 2007; 2011) | 1,344 |
| Homology sequence-based | GERP;  GERP++ | Genomic Evolutionary Rate Profiling | SNVs | <http://mendel.stanford.edu/sidowlab/downloads/gerp/index.html> | Stand-alone | July, 2005 | May 22, 2011 | - | MSA, maximum likelihood, dynamic programming | Sequence homology, phylogenetic tree | Human genome and 33 other mammalian species | RS score | - | - | - | (Cooper et al., 2005; Davydov et al., 2010) | 1,825 |
| Homology sequence-based | phastcons | - | Genome-wide | <http://compgen.cshl.edu/phast/> | Stand-alone | Aug., 2005 | Feb. 11, 2014 | C | Two-state phylogenetic hidden Markov model (phylo-HMM) and EM algorithm | Sequence homology, phylogenetic tree | MSA of 5 vertebrate species, 4 insect species, 2 species of Caenorhabditis, and 7 species of Saccharomyces | Conservation score | Conserved/ non-conserved | - | - | (Siepel et al., 2005) | 2,432 |
| Homology sequence-based | PROVEAN | Protein Variation Effect Analyzer | nsSNPs, inframe-indels | <http://provean.jcvi.org/> | Web and stand-alond | Oct., 2012 | Jan. 30, 2015 | - | MSA | Sequence homology | Homologous sequences from the NCBI NR protein database | PROVEAN score | Deleterious/ neutral | <-2.282;  ≥-2.282 | SNVs: 20,821 disease variants and 36,825 common SNPs from UniProt and 729, 171, and 138 indels | (Choi et al., 2012; Choi and Chan, 2015) | 2,297 |
| Homology sequence-based | PANTHER | Protein ANalysis THrough Evolutionary Relationships | nsSNPs | <http://www.pantherdb.org/> | Web and stand-alond | Oct., 2004 | Feb. 22, 2022 | - | HMM | Sequence homology, phylogenetic tree | Multiple sequence alignment | Substitution position-specific evolutionary conservation (subPSEC) score | Deleterious/ neutral | <0;  ≥0 | 12,519 disease-associated SNPs from HGMD; 10,586 neutral SNPs from dbSNP | (Thomas et al., 2003; Thomas and Kejariwal, 2004; Thomas et al., 2006; Mi et al., 2021) | 2,655 |
| Homology sequence-based | PhD-SNP | Predictor of human Deleterious Single Nucleotide Polymorphisms | nsSNPs | <https://snps.biofold.org/phd-snp/phd-snp.html> | Web and stand-alond | Nov., 2006 | Jan. 18, 2010 | - | SVM | 40 values: 20 residue types indicating mutation, 20 environment values, and 4 addition values | Multiple sequence alignment | O[D] | Disease/ neutral | [0,0.5);  [0.5,1] | Disease-related: 12,944 nsSNPs; neutral: 8,241 nsSNPs from SwissProt | (Capriotti et al., 2006) | 526 |
| Homology sequence-based | EVE | Evolutionary model of variant effect | Protein mutations | <https://evemodel.org/> | Web and stand-alone | Sept., 2021 | - | Python | Deep generative model, a Bayesian variational autoencoder (VAE), a two-component global-local mixture of Gaussian mixture models | Homology sequence | - | EVE score | Benign/ uncertain/ pathogenic | - | 3,219 proteins from ClinVar | (Frazer et al., 2021) | 12 (PubMed) |
| Homology sequence-based | LRT | Likelihood ratio test | Coding SNVs | - | Pre-computed results | Sept., 2009 | - | - | Likelihood-ratio test | MSA | - | Log-likelihood ratio (LLR) | Deleterious/  neutral/  unknown | - | - | (Chun and Fay, 2009) | 651 |
| Homology sequence-based | SiPhy | SIte-specific PHYlogenetic analysis | SNPs | <https://portals.broadinstitute.org/genome_bio/siphy/> | Stand-alone | June, 2009 | - | JS | Two-state HMM and site-specific nucleotide substitution patterns with window-based log-odds ratio | MSA, phylogenetic tree | - | Log-odds (LO) score | Conservative/ non-conservative | - | - | (Garber et al., 2009)19478016 | 206 |
| Homology sequence-based | DeepSequence | - | SNPs | <https://github.com/debbiemarkslab/DeepSequence> | Stand-alone | Oct., 2018 | - | Python | Deep generative model, two hidden layers NN, nonlinear latent-variable model VAE | HMM-MSA | - | Log-ratio | Conservation/ frequency/ interaction | - | - | (Riesselman et al., 2018) | 117 |
| Homology sequence-based | EVmutation | - | SNPs, indels | <http://evmutation.org/> | Stand-alone and precomputed results | Jan., 2017 | - | C, python | Probabilistic approach (Markov Random field), or a Potts model | HMM-MSA | - | Evolutionary statistical energy difference | Deleterious/ neutral/ beneficial | <0;  =0;  >0 | 34 datasets from 29 experiments in 21 proteins and a tRNA gene | (Hopf et al., 2017) | 224 |
| Homology sequence-based | phyloP | - | Nucleotide substitution rates | <http://compgen.cshl.edu/phast/> | Stand-alone | Jan., 2010 | Feb. 11, 2014 | C | Four statistical, phylogenetic test | Sequence homology, phylogenetic tree | 36 mammalian species | P-value | Conservation/neutral | - | - | (Pollard et al., 2010) | 1,191 |
| Structural-based | MAESTRO | Multi AgEnt STability pRedictiOn | AA substitutions | <https://pbwww.services.came.sbg.ac.at/?page_id=416> | Web and stand-alone | April, 2015 | Jan. 6, 2016 | - | Multi-agent prediction system | Statistical scoring functions and protein properties | PDB set and 5 datasets derived from the ProTherm database | MAESTRO score | Destabilization/  stability | >0;  <0 | 2 datasets | (Laimer et al., 2015) | 132 |
| Structural-based | PoPMuSiC-2.0 | - | AA substitutions | <https://soft.dezyme.com/query/create/pop> | Web | 1997 | June, 2014 | - | Neural network | 5 groups of potentials | Dataset extracted from ProTherm database with experimentaltest on stability impact includes 2648 mutations | ΔΔG predictions | Destabilization/  stability | >0;  <0 | - | (Dehouck et al., 2009) | 259 |
| Structural-based | SDM | Site Directed Mutator | AA substitutions | <http://marid.bioc.cam.ac.uk/sdm2/help> | Web | April, 2008 | May, 2017 | - | Statistical potential energy function | ESSTs from TOCCATA database with 2 further structural parameters based on residue-occluded packing density (OSP) and residue depth | - | Stability difference score | Destabilization/  stability | <0;  >0 | 4 dataset from ProTherm and literatures | (Worth et al., 2011; Pandurangan et al., 2017) | 555 |
| Multiple features | PolyPhen2 | Polymorphism Phenotyping v2 | Missense mutations | <http://genetics.bwh.harvard.edu/pph2/> | Web and stand-alone | April, 2010 | June 21, 2021 | - | Naïve bayes classifier | 8 sequence-based and 3 structure-based predictive features | - | Naïve Bayes probabilistic score | Probably damaging/  possibly damaging/  benign | (0.85,1];  (0.15,0.85];  [0,0.15] | - | (Adzhubei et al., 2010) | 8,595 |
| Multiple features | MutPred; MutPred2 | - | AA subsititutions | <http://mutpred.mutdb.org/> | Web and stand-alone | Nov., 2009 | Nov. 20, 2020 | - | RF or SVM; bagged ensemble of feed-forward neural networks | 7 structural and 7 functional properties; 53 structural and functional protein properties | Disease-associated mutations from 4 datasets (Cancer, Kinase, HGMD, Swiss-Port); polymorphic mutations from Swiss-Prot; 53,180 pathogenic and 206,946 putatively neutral variants obtained from the HGMD, SwissVar, dbSNP, and interspecies pairwise alignments | General score & property score | Pathogenic/ non-pathogenic | Under FPR of 10%:  [0,0.68); [0.68,1].  Under FPR of 5%:  [0,0.8);  [0.8,1] | ClinVar and UniProt | (Li et al., 2009; Pejaver et al., 2020) | 608 |
| Multiple features | VEST | Variant Effect Scoring Tool | Missense variants | <https://karchinlab.org/apps/appVest.html> | Stand-alone | May 2013 | Jan. 5, 2014 | - | RF | 86 quantitative features | 47,724 disease mutations from HGMD; 45,818 putatively neutral missense variants from the ESP6500 | - | - | - | PolyPhen2.2.2 training set release dated December 2011 | (Carter et al., 2013) | 227 |
| Multiple features | SNAP;  SNAP2 | Screening for non-acceptable polymorphisms | nsSNPs | <https://www.rostlab.org/services/SNAP/> | Web and stand-alond | May, 2007 | June 18, 2015 | Perl | Neural network | Explicit PSI-BLAST frequency profile, and structural features | 40,641 damaging nsSNPs and 14,334 neutral nsSNPs from PMD; 26,840 neutral pseudo-mutants from SWISS-PORT | Neural network based calssifier | Disease/ neutral | - | 4,041 LacI mutants, 2,015 Lysozyme mutants, and 336 HIV-1 protease mutants | (Bromberg and Rost, 2007; Hecht et al., 2015) | 809 |
| Multiple features | MAPP | Multivariate Analysis of Protein Polymorphism | Missense variants | <http://mendel.stanford.edu/SidowLab/downloads/MAPP/index.html> | Stand-alone | June, 2005 | - | JS | Principal component transformation | MSA, phylogenetic tree, structural features | - | Impact scores | Wild-type function; moderately deleterious; strong loss of function | >0;  =0;  <0 | Four mutagenesis experiments on HIV protease, LacI, HIV RT, T4 lysozyme | (Stone and Sidow, 2005)15965030 | 252 |
| Multiple features | SNPs3D | - | nsSNPs | <http://www.snps3d.org/> | Web | March, 2006 | Oct. 29, 2008 | JS | SVM | Sequence profile and structure | 9,625 variants from HGMD; 29,485 variants from dbSNP v124 | SVM scores | Deleterious/ neutral | <0;  >0 | - | (Yue et al., 2006) | 352 |
| Multiple features | ENTPRISE | ENTropy and PRedIcted protein StructurE | nsSNPs | <http://cssb2.biology.gatech.edu/ENTPRISE/> | Web and pre-computed results | March, 2016 | - | - | Boosted tree regression | 81 features including entropy, 20 wildtype amino acid, 20 mutant amino acid, domain composition of 20 AA, contacting composition of 20 AA | Datasets in PredictSNP | ENTPRISE scores | Disease-associated/  neutral | >0.45;  <0.45 | 1KG and VariSNP sets; cancer-driven gene sets from COSMIC, TCGA, COBR | (Zhou et al., 2016) | 17 |
| Multiple features | ELASPIC | Ensemble Learning Approach for Stability Prediction of Interface and Core mutations | Domain nsSNPs | <http://elaspic.kimlab.org/> | Web and stand-alone | Sept., 2014 | May 15, 2016 | Python | Stochastic Gradient Boosting of Decision Trees (SGB-DT) | 3 categories including 75 features: sequence features, energy features and molecular features | 3,463 variants from ProTherm; 873 variants from SKEMPI | △△G_DT_ | - | - | 10,071 disease-associated variants from OMIM, 15,951 cancer-driver mutations from COSMIC; 1,206 neutral variants from HapMap | (Berliner et al., 2014; Witvliet et al., 2016) | 62 |
| Multiple features | MutationTaster; MutationTaster2; MutationTaster2021 | - | SNPs, indels and splicing-site | <https://www.mutationtaster.org/> | Web | Aug., 2010 | April 24, 2021 | Perl | MutationTaster/MutationTaster2: naïve Bayes classifier; MutationTaster2021: RF | Genetic features and protein features | >100,000 disease mutations from HGMD; >6,000,000 validated neutral variants from 1KGP and BIOBASE | Probability value/RF classification | Deleterious/  benign | - | 1,100 common polymorphisms and 1,100 known disease mutations | (Schwarz et al., 2010; Schwarz et al., 2014; Steinhaus et al., 2021) | 4,215 |
| Multiple features | SNPs&GO | classifying human SNPs by including GO | nsSNPs | <https://snps-and-go.biocomp.unibo.it/snps-and-go/index.html> | Web | Aug., 2009 | - | - | SVM | 52 features | 16,330 disease-related, 17,432 neutral variants from Swiss-Prot | O[D] | Disease-related/  neutral | [0,0.5);  [0.5,1] | - | (Calabrese et al., 2009) | 393 |
| Multiple features | PON-P2 | Pathogenic-or-Not-Pipeline | amino acid subsititutions | <http://structure.bmc.lu.se/PON-P2/> | Web and stand-alone | Feb., 2015 | March 24, 2016 | R | RF | 8 features including amino acid, GO, conservation, functional and structural annotations | 14,610 pathogenic and 17,393 neutral variants from VariBench | RF classifiers | Pathogenic/ unknown classes/ neutral | - | 501 pathogenic and 571 neutral variants from MutationTaster2 dataset | (Niroula et al., 2015) | 123 |
| Meta-predictor | CADD | Combined Annotation Dependent Depletion | SNP, indels | <https://cadd.gs.washington.edu/> | Web and stand-alone | March, 2014 | Feb. 22, 2021 | - | SVM with a linear kernal | 949 features : containing conservation metrics, regulatory, transcription information and protein-level scores | 13,141,299 SNVs, 627,071 insertions and 926,968 deletions | C-score | - | - | - | (Kircher et al., 2014) | 3,353 |
| Meta-predictor | DANN | - | SNPs | <https://cbcl.ics.uci.edu/public_data/DANN/> | Web and stand-alone | March, 2015 | - | Python | Neural network | Same as CADD | Training data used in CADD, including 16,627,775 "observed" variants and 49,407,057 "simulated" variants | Neural network based calssifier | Pathogenic/ benign | - | 10,000 pathogenic mutations from ClinVar; 10,000 benign mutations ESP | (Quang et al., 2015) | 476 |
| Meta-predictor | REVEL | Rare Exome Variant Ensemble Learner | Missense mutations | <https://sites.google.com/site/revelgenomics/> | Stand-alone | Oct., 2016 | May 3, 2021 | R | RF | 18 individual pathogenicity prediction scores from 13 tools | 6,182 disease variants from HGMD; 281,972 neutral missense exome sequencing variants from ESP, 1KGP | REVEL score | Pathogenic/ neutral | - | Dataset 1: 935 disease variants from SwissVar; 141,051 neutral variants; Dataset 2: 1,953 P/LP; 2,406 B/LB variants from ClinVar | (Ioannidis et al., 2016) | 644 |
| Meta-predictor | Meta-SNP | - | nsSNPs | <https://snps.biofold.org/meta-snp/> | Web and stand-alond | May, 2012 | - | Python | RF | 4 predictors results and 4 elements extracted from PhD-SNP | SwissVar database October 2009 release (SV-2009) | Probability of disease-related | Disease/ polymorphism | (0.5,1];  (0,0.5] | SwissVar (Feb., 2012 release) | (Capriotti et al., 2013)23819846 | 124 |
| Meta-predictor | CONDEL;  CONDEL 2.0 | CONsensus DELeteriousness | nsSNPs | <https://bbglab.irbbarcelona.org/fannsdb/query/condel> | Web and pre-computed results | April, 2011 | 2014 | Perl | Weighted average of the normalized scores of five methods | Prediction results from 5 tools | 12,405 deleterious, 8,257 common SNVs from HumVar;: 3,155 disease-related mutations, 6,321 orthologous changes in proteins from HumDiv | WAS | Deleterious/ neutral | (0.522,1]; [0,0.522] | COSMIC: 12,640 deleterious; IARC TP53 database: 2,312 deleterious | (Gonzalez-Perez and Lopez-Bigas, 2011) | 561 |
| Meta-predictor | MutScore | - | Missense mutations | <https://mutscore-wgt7hvakhq-ew.a.run.app/> | Web and stand-alone | March, 2022 | - | R | RF | 18 features | ClinVar and gnomAD dataset r.2.1.1 | MutScore score | Pathogenic/ benign | - | - | (Quinodoz et al., 2022) | 2 (PubMed) |
| Meta-predictor | BayesDel | - | Coding and non-coding variants, single nucleotide variants and small indels | <https://fengbj-laboratory.org/> | Stand-alone and pre-computed results | Dec., 2016 | Aug. 24, 2017 | - | Naïve bayes classifier | PolyPhen2, SIFT, FATHMM, LRT, Mutation Taster, Mutation Assessor, PhyloP, GERP++, and SiPhy | P/LP variants from ClinVar and disease variants in UniProtKB; neutral variants from dbSNP, 1GKP, Exac, the ALSPAC and TWINSUK cohorts in the UK10K Project, excluding the above pathogenic variants | Deleteriousness score | Pathogenic/ non-pathogenic | - | - | (Feng, 2017) | 31 |
| Meta-predictor | MetaLR | - | nsSNPs | <https://sites.google.com/site/jpopgen/dbNSFP> | Pre-computed results | April, 2015 | - | R | Logistic regression | 10 features include prediction scores | Uniprot, CHARGE sequencing project and VariBench dataset | LR scores | Deleterious/ neutral | [0.5,1];  [0,0.5) | 10,164 rare and novel neutral singleton mutations from CHARGE sequencing project | (Dong et al., 2015) | 563 |
| Meta-predictor | MetaSVM | - | nsSNPs | <https://sites.google.com/site/jpopgen/dbNSFP> | Pre-computed results | April, 2015 | - | R | SVM | 10 features include prediction scores | Uniprot, CHARGE sequencing project and VariBench dataset | SVM scores | Deleterious/ neutral | ≥0;  <0 | 10,164 rare and novel neutral singleton mutations from CHARGE sequencing project | (Dong et al., 2015) | 563 |
| Meta-predictor | M-CAP | The Mendelian Clinically Applicable Pathogenicity | Missense variants | <http://bejerano.stanford.edu/mcap/> | Web and stand-alone | Dec., 2016 | March 25, 2019 | Python | Gradient boosting trees (GBDT) | 318 features | 12,418 Disease-mutation variants from HGMD; 3,137,919 neutral mutations from ExAC. | M-CAP score | Pathogenic/ benign | >0.025 | 17 *BRCA1*, 10 *BRCA2*, 39 *CFTR* and 19 *MLL2* pathogenic mutations from Eigen website | (Jagadeesh et al., 2016) | 389 |
| Meta-predictor | PredictSNP;  PredictSNP2 | - | Regulatory, splicing, missense, synonymous and nonsense variants | <https://loschmidt.chemi.muni.cz/predictsnp2/> | Web and precomputed results | Jan., 2014 | May 25, 2016 | - | Confidence-weighted majority vote approach | Prediction scores from 6 pre-existing tools | 33,356 disease-associated variants from ClinVar; 5,591,761 neutral variants from VariSNP | PredictSNP2 consensus score | Deleterious/ neutral | (0,1];  [-1,0] | 12,050 disease-associated variants in complex disease datasets and 142,722 variants in somatic cancer datasets | (Bendl et al., 2014) | 470 |
| MAF as features | ClinPred | - | nsSNPs | <https://sites.google.com/site/clinpred/> | Pre-computed results | Oct., 2018 | - | - | Combines random forest and gradient boosting models | MAF from gnomAD, and 16 individual prediction scores | ClinVar | ClinPred scores | Pathogenic/ benign | (0.5,1];  [0.0.5] | 437 LoF and 1464 functional variants from *BRCA1* dataset | (Alirezaie et al., 2018) | 60 |
| MAF as features | CAPICE | Consequence-Agnostic prediction of Pathogenicity Interpretation of Clinical Exome variations | SNP, indels | <https://capice.molgeniscloud.org/> | Web, stand-alone and precomputed results | Jan., 2017 | Feb. 17, 2022 | Python | Gradient boosting on decision trees (GBDT) | 92 features | 40,681 pathogenic and 293,920 neutral variants from ClinVar, VKGL and literatures | CAPICE classification score | Pathogenic/  benign | >0.02 | Set1: 60,699 neutral variants from literature; Set2: 14,426,914 neutral variants from GoNL | (Li et al., 2020) | 8 |
| MAF as features | UMD-predictor | - | SNPs | [http://umd-predictor.eu](http://umd-predictor.eu/) | Stand-alone and precomputed results | June, 2009 | Feb. 22, 2016 | PHP, JS and html | Empirical statistic equation | 7 features | - | UMDscore | Polymorphism/  probable polymorphism/  probable pathogenic/  pathogenic | [0,50); [50,65); [65,75); [75,100] | Dataset 1: Varibench; dataset 2: UniProt; dataset 3: Clinvar; dataset 4: PredictSNP | (Frederic et al., 2009; Salgado et al., 2016) | 150 |
| MAF as features | MISTIC | MISsense deleTeriousness predICtor | Missense | <http://lbgi.fr/mistic/> | Web, stand-alone and precomputed results | July, 2020 | - | Python | Soft Voting system based on weighted average of Random Forest and Logistic regression models | 113 features from multi-ethnic MAF values, functional and conservation measures, scores from prediction tools | 11,190 deleterious missense from ClinVar and HGMD Pro databases; 11,190 rare neutral variants from gnomAD | Scores | Deleterious/  benign | >0.5;  <0.5 | 6 datasets including ClinVar, DoCM, gnomAD, UK10K, SweGen, WesternAsia | (Chennen et al., 2020) | 9 |
| MAF as features | LEAP | Learning from Evidence to Assess Pathogenicity | Missense | <https://www.ncbi.nlm.nih.gov/clinvar/submitters/505849/> | Pre-computed results | June, 2020 | Jan., 2022 | Python | RF and logistic regression | 245 features in total include functional, splicing impact, location, MAF, aggregated individual-level information | 19,624 missense variants classified to P/LP, VUS, B/LB according to ACMG guidelines | ML classification | - | - | - | (Lai et al., 2020) | 8 |

*P:pathogenic, LP: likely pathogenic, B: benign; LB: likely benign; VUS: Variant of Uncertain Significance

Adzhubei, I.A., Schmidt, S., Peshkin, L., Ramensky, V.E., Gerasimova, A., Bork, P., et al. (2010). A method and server for predicting damaging missense mutations. *Nat Methods* 7(4)**,** 248-249. doi: 10.1038/nmeth0410-248.

Alirezaie, N., Kernohan, K.D., Hartley, T., Majewski, J., and Hocking, T.D. (2018). ClinPred: Prediction Tool to Identify Disease-Relevant Nonsynonymous Single-Nucleotide Variants. *Am J Hum Genet* 103(4)**,** 474-483. doi: 10.1016/j.ajhg.2018.08.005.

Bendl, J., Stourac, J., Salanda, O., Pavelka, A., Wieben, E.D., Zendulka, J., et al. (2014). PredictSNP: robust and accurate consensus classifier for prediction of disease-related mutations. *PLoS Comput Biol* 10(1)**,** e1003440. doi: 10.1371/journal.pcbi.1003440.

Berliner, N., Teyra, J., Colak, R., Garcia Lopez, S., and Kim, P.M. (2014). Combining structural modeling with ensemble machine learning to accurately predict protein fold stability and binding affinity effects upon mutation. *PLoS One* 9(9)**,** e107353. doi: 10.1371/journal.pone.0107353.

Bromberg, Y., and Rost, B. (2007). SNAP: predict effect of non-synonymous polymorphisms on function. *Nucleic Acids Res* 35(11)**,** 3823-3835. doi: 10.1093/nar/gkm238.

Calabrese, R., Capriotti, E., Fariselli, P., Martelli, P.L., and Casadio, R. (2009). Functional annotations improve the predictive score of human disease-related mutations in proteins. *Hum Mutat* 30(8)**,** 1237-1244. doi: 10.1002/humu.21047.

Capriotti, E., Altman, R.B., and Bromberg, Y. (2013). Collective judgment predicts disease-associated single nucleotide variants. *BMC Genomics* 14 Suppl 3**,** S2. doi: 10.1186/1471-2164-14-S3-S2.

Capriotti, E., Calabrese, R., and Casadio, R. (2006). Predicting the insurgence of human genetic diseases associated to single point protein mutations with support vector machines and evolutionary information. *Bioinformatics* 22(22)**,** 2729-2734. doi: 10.1093/bioinformatics/btl423.

Carter, H., Douville, C., Stenson, P.D., Cooper, D.N., and Karchin, R. (2013). Identifying Mendelian disease genes with the variant effect scoring tool. *BMC Genomics* 14 Suppl 3**,** S3. doi: 10.1186/1471-2164-14-S3-S3.

Chennen, K., Weber, T., Lornage, X., Kress, A., Bohm, J., Thompson, J., et al. (2020). MISTIC: A prediction tool to reveal disease-relevant deleterious missense variants. *PLoS One* 15(7)**,** e0236962. doi: 10.1371/journal.pone.0236962.

Choi, Y., and Chan, A.P. (2015). PROVEAN web server: a tool to predict the functional effect of amino acid substitutions and indels. *Bioinformatics* 31(16)**,** 2745-2747. doi: 10.1093/bioinformatics/btv195.

Choi, Y., Sims, G.E., Murphy, S., Miller, J.R., and Chan, A.P. (2012). Predicting the functional effect of amino acid substitutions and indels. *PLoS One* 7(10)**,** e46688. doi: 10.1371/journal.pone.0046688.

Chun, S., and Fay, J.C. (2009). Identification of deleterious mutations within three human genomes. *Genome Res* 19(9)**,** 1553-1561. doi: 10.1101/gr.092619.109.

Cooper, G.M., Stone, E.A., Asimenos, G., Program, N.C.S., Green, E.D., Batzoglou, S., et al. (2005). Distribution and intensity of constraint in mammalian genomic sequence. *Genome Res* 15(7)**,** 901-913. doi: 10.1101/gr.3577405.

Davydov, E.V., Goode, D.L., Sirota, M., Cooper, G.M., Sidow, A., and Batzoglou, S. (2010). Identifying a high fraction of the human genome to be under selective constraint using GERP++. *PLoS Comput Biol* 6(12)**,** e1001025. doi: 10.1371/journal.pcbi.1001025.

Dehouck, Y., Grosfils, A., Folch, B., Gilis, D., Bogaerts, P., and Rooman, M. (2009). Fast and accurate predictions of protein stability changes upon mutations using statistical potentials and neural networks: PoPMuSiC-2.0. *Bioinformatics* 25(19)**,** 2537-2543. doi: 10.1093/bioinformatics/btp445.

Dong, C., Wei, P., Jian, X., Gibbs, R., Boerwinkle, E., Wang, K., et al. (2015). Comparison and integration of deleteriousness prediction methods for nonsynonymous SNVs in whole exome sequencing studies. *Hum Mol Genet* 24(8)**,** 2125-2137. doi: 10.1093/hmg/ddu733.

Feng, B.J. (2017). PERCH: A Unified Framework for Disease Gene Prioritization. *Hum Mutat* 38(3)**,** 243-251. doi: 10.1002/humu.23158.

Frazer, J., Notin, P., Dias, M., Gomez, A., Min, J.K., Brock, K., et al. (2021). Disease variant prediction with deep generative models of evolutionary data. *Nature* 599(7883)**,** 91-95. doi: 10.1038/s41586-021-04043-8.

Frederic, M.Y., Lalande, M., Boileau, C., Hamroun, D., Claustres, M., Beroud, C., et al. (2009). UMD-predictor, a new prediction tool for nucleotide substitution pathogenicity -- application to four genes: FBN1, FBN2, TGFBR1, and TGFBR2. *Hum Mutat* 30(6)**,** 952-959. doi: 10.1002/humu.20970.

Garber, M., Guttman, M., Clamp, M., Zody, M.C., Friedman, N., and Xie, X. (2009). Identifying novel constrained elements by exploiting biased substitution patterns. *Bioinformatics* 25(12)**,** i54-62. doi: 10.1093/bioinformatics/btp190.

Gonzalez-Perez, A., and Lopez-Bigas, N. (2011). Improving the assessment of the outcome of nonsynonymous SNVs with a consensus deleteriousness score, Condel. *Am J Hum Genet* 88(4)**,** 440-449. doi: 10.1016/j.ajhg.2011.03.004.

Hecht, M., Bromberg, Y., and Rost, B. (2015). Better prediction of functional effects for sequence variants. *BMC Genomics* 16 Suppl 8**,** S1. doi: 10.1186/1471-2164-16-S8-S1.

Hopf, T.A., Ingraham, J.B., Poelwijk, F.J., Scharfe, C.P., Springer, M., Sander, C., et al. (2017). Mutation effects predicted from sequence co-variation. *Nat Biotechnol* 35(2)**,** 128-135. doi: 10.1038/nbt.3769.

Ioannidis, N.M., Rothstein, J.H., Pejaver, V., Middha, S., McDonnell, S.K., Baheti, S., et al. (2016). REVEL: An Ensemble Method for Predicting the Pathogenicity of Rare Missense Variants. *Am J Hum Genet* 99(4)**,** 877-885. doi: 10.1016/j.ajhg.2016.08.016.

Jagadeesh, K.A., Wenger, A.M., Berger, M.J., Guturu, H., Stenson, P.D., Cooper, D.N., et al. (2016). M-CAP eliminates a majority of variants of uncertain significance in clinical exomes at high sensitivity. *Nat Genet* 48(12)**,** 1581-1586. doi: 10.1038/ng.3703.

Kircher, M., Witten, D.M., Jain, P., O'Roak, B.J., Cooper, G.M., and Shendure, J. (2014). A general framework for estimating the relative pathogenicity of human genetic variants. *Nat Genet* 46(3)**,** 310-315. doi: 10.1038/ng.2892.

Lai, C., Zimmer, A.D., O'Connor, R., Kim, S., Chan, R., van den Akker, J., et al. (2020). LEAP: Using machine learning to support variant classification in a clinical setting. *Hum Mutat* 41(6)**,** 1079-1090. doi: 10.1002/humu.24011.

Laimer, J., Hofer, H., Fritz, M., Wegenkittl, S., and Lackner, P. (2015). MAESTRO--multi agent stability prediction upon point mutations. *BMC Bioinformatics* 16**,** 116. doi: 10.1186/s12859-015-0548-6.

Li, B., Krishnan, V.G., Mort, M.E., Xin, F., Kamati, K.K., Cooper, D.N., et al. (2009). Automated inference of molecular mechanisms of disease from amino acid substitutions. *Bioinformatics* 25(21)**,** 2744-2750. doi: 10.1093/bioinformatics/btp528.

Li, S., van der Velde, K.J., de Ridder, D., van Dijk, A.D.J., Soudis, D., Zwerwer, L.R., et al. (2020). CAPICE: a computational method for Consequence-Agnostic Pathogenicity Interpretation of Clinical Exome variations. *Genome Med* 12(1)**,** 75. doi: 10.1186/s13073-020-00775-w.

Mi, H., Ebert, D., Muruganujan, A., Mills, C., Albou, L.P., Mushayamaha, T., et al. (2021). PANTHER version 16: a revised family classification, tree-based classification tool, enhancer regions and extensive API. *Nucleic Acids Res* 49(D1)**,** D394-D403. doi: 10.1093/nar/gkaa1106.

Ng, P.C., and Henikoff, S. (2001). Predicting deleterious amino acid substitutions. *Genome Res* 11(5)**,** 863-874. doi: 10.1101/gr.176601.

Ng, P.C., and Henikoff, S. (2003). SIFT: Predicting amino acid changes that affect protein function. *Nucleic Acids Res* 31(13)**,** 3812-3814. doi: 10.1093/nar/gkg509.

Niroula, A., Urolagin, S., and Vihinen, M. (2015). PON-P2: prediction method for fast and reliable identification of harmful variants. *PLoS One* 10(2)**,** e0117380. doi: 10.1371/journal.pone.0117380.

Pandurangan, A.P., Ochoa-Montano, B., Ascher, D.B., and Blundell, T.L. (2017). SDM: a server for predicting effects of mutations on protein stability. *Nucleic Acids Res* 45(W1)**,** W229-W235. doi: 10.1093/nar/gkx439.

Pejaver, V., Urresti, J., Lugo-Martinez, J., Pagel, K.A., Lin, G.N., Nam, H.J., et al. (2020). Inferring the molecular and phenotypic impact of amino acid variants with MutPred2. *Nat Commun* 11(1)**,** 5918. doi: 10.1038/s41467-020-19669-x.

Pollard, K.S., Hubisz, M.J., Rosenbloom, K.R., and Siepel, A. (2010). Detection of nonneutral substitution rates on mammalian phylogenies. *Genome Res* 20(1)**,** 110-121. doi: 10.1101/gr.097857.109.

Quang, D., Chen, Y., and Xie, X. (2015). DANN: a deep learning approach for annotating the pathogenicity of genetic variants. *Bioinformatics* 31(5)**,** 761-763. doi: 10.1093/bioinformatics/btu703.

Quinodoz, M., Peter, V.G., Cisarova, K., Royer-Bertrand, B., Stenson, P.D., Cooper, D.N., et al. (2022). Analysis of missense variants in the human genome reveals widespread gene-specific clustering and improves prediction of pathogenicity. *Am J Hum Genet* 109(3)**,** 457-470. doi: 10.1016/j.ajhg.2022.01.006.

Reva, B., Antipin, Y., and Sander, C. (2007). Determinants of protein function revealed by combinatorial entropy optimization. *Genome Biol* 8(11)**,** R232. doi: 10.1186/gb-2007-8-11-r232.

Reva, B., Antipin, Y., and Sander, C. (2011). Predicting the functional impact of protein mutations: application to cancer genomics. *Nucleic Acids Res* 39(17)**,** e118. doi: 10.1093/nar/gkr407.

Riesselman, A.J., Ingraham, J.B., and Marks, D.S. (2018). Deep generative models of genetic variation capture the effects of mutations. *Nat Methods* 15(10)**,** 816-822. doi: 10.1038/s41592-018-0138-4.

Salgado, D., Desvignes, J.P., Rai, G., Blanchard, A., Miltgen, M., Pinard, A., et al. (2016). UMD-Predictor: A High-Throughput Sequencing Compliant System for Pathogenicity Prediction of any Human cDNA Substitution. *Hum Mutat* 37(5)**,** 439-446. doi: 10.1002/humu.22965.

Schwarz, J.M., Cooper, D.N., Schuelke, M., and Seelow, D. (2014). MutationTaster2: mutation prediction for the deep-sequencing age. *Nat Methods* 11(4)**,** 361-362. doi: 10.1038/nmeth.2890.

Schwarz, J.M., Rodelsperger, C., Schuelke, M., and Seelow, D. (2010). MutationTaster evaluates disease-causing potential of sequence alterations. *Nat Methods* 7(8)**,** 575-576. doi: 10.1038/nmeth0810-575.

Shihab, H.A., Gough, J., Cooper, D.N., Day, I.N., and Gaunt, T.R. (2013a). Predicting the functional consequences of cancer-associated amino acid substitutions. *Bioinformatics* 29(12)**,** 1504-1510. doi: 10.1093/bioinformatics/btt182.

Shihab, H.A., Gough, J., Cooper, D.N., Stenson, P.D., Barker, G.L., Edwards, K.J., et al. (2013b). Predicting the functional, molecular, and phenotypic consequences of amino acid substitutions using hidden Markov models. *Hum Mutat* 34(1)**,** 57-65. doi: 10.1002/humu.22225.

Shihab, H.A., Gough, J., Mort, M., Cooper, D.N., Day, I.N., and Gaunt, T.R. (2014). Ranking non-synonymous single nucleotide polymorphisms based on disease concepts. *Hum Genomics* 8**,** 11. doi: 10.1186/1479-7364-8-11.

Siepel, A., Bejerano, G., Pedersen, J.S., Hinrichs, A.S., Hou, M., Rosenbloom, K., et al. (2005). Evolutionarily conserved elements in vertebrate, insect, worm, and yeast genomes. *Genome Res* 15(8)**,** 1034-1050. doi: 10.1101/gr.3715005.

Sim, N.L., Kumar, P., Hu, J., Henikoff, S., Schneider, G., and Ng, P.C. (2012). SIFT web server: predicting effects of amino acid substitutions on proteins. *Nucleic Acids Res* 40(Web Server issue)**,** W452-457. doi: 10.1093/nar/gks539.

Steinhaus, R., Proft, S., Schuelke, M., Cooper, D.N., Schwarz, J.M., and Seelow, D. (2021). MutationTaster2021. *Nucleic Acids Res* 49(W1)**,** W446-W451. doi: 10.1093/nar/gkab266.

Stone, E.A., and Sidow, A. (2005). Physicochemical constraint violation by missense substitutions mediates impairment of protein function and disease severity. *Genome Res* 15(7)**,** 978-986. doi: 10.1101/gr.3804205.

Thomas, P.D., Campbell, M.J., Kejariwal, A., Mi, H., Karlak, B., Daverman, R., et al. (2003). PANTHER: a library of protein families and subfamilies indexed by function. *Genome Res* 13(9)**,** 2129-2141. doi: 10.1101/gr.772403.

Thomas, P.D., and Kejariwal, A. (2004). Coding single-nucleotide polymorphisms associated with complex vs. Mendelian disease: evolutionary evidence for differences in molecular effects. *Proc Natl Acad Sci U S A* 101(43)**,** 15398-15403. doi: 10.1073/pnas.0404380101.

Thomas, P.D., Kejariwal, A., Guo, N., Mi, H., Campbell, M.J., Muruganujan, A., et al. (2006). Applications for protein sequence-function evolution data: mRNA/protein expression analysis and coding SNP scoring tools. *Nucleic Acids Res* 34(Web Server issue)**,** W645-650. doi: 10.1093/nar/gkl229.

Vaser, R., Adusumalli, S., Leng, S.N., Sikic, M., and Ng, P.C. (2016). SIFT missense predictions for genomes. *Nat Protoc* 11(1)**,** 1-9. doi: 10.1038/nprot.2015.123.

Witvliet, D.K., Strokach, A., Giraldo-Forero, A.F., Teyra, J., Colak, R., and Kim, P.M. (2016). ELASPIC web-server: proteome-wide structure-based prediction of mutation effects on protein stability and binding affinity. *Bioinformatics* 32(10)**,** 1589-1591. doi: 10.1093/bioinformatics/btw031.

Worth, C.L., Preissner, R., and Blundell, T.L. (2011). SDM--a server for predicting effects of mutations on protein stability and malfunction. *Nucleic Acids Res* 39(Web Server issue)**,** W215-222. doi: 10.1093/nar/gkr363.

Yue, P., Melamud, E., and Moult, J. (2006). SNPs3D: candidate gene and SNP selection for association studies. *BMC Bioinformatics* 7**,** 166. doi: 10.1186/1471-2105-7-166.

Zhou, H., Gao, M., and Skolnick, J. (2016). ENTPRISE: An Algorithm for Predicting Human Disease-Associated Amino Acid Substitutions from Sequence Entropy and Predicted Protein Structures. *PLoS One* 11(3)**,** e0150965. doi: 10.1371/journal.pone.0150965.
